# Diagnostic accuracy of ovarian cancer using convolutional neural network: a systematic review and meta-analysis

**DOI:** 10.1186/s12911-026-03462-9

**Published:** 2026-04-18

**Authors:** Leila Allahqoli, Atieh Karimzadeh, Ali Kazemi Abadi, Seyedeh Zahra Aghamohammadi, Sevil Hakimi, Azam Rahmani, Arezoo Fallahi, Hamid Salehiniya, Antonio Simone Laganà, Akshaya Srikanth Bhagavathula, Mohammadmatin Ghiyasvand

**Affiliations:** 1 Faculty of Health Sciences, Cyprus International University, Nicosia, North Cyprus Cyprus; 2 School of Medicine, Iran University of Medical Sciences, Tehran, Iran; 3 School of Medicine, Islamic Azad University of Medical Sciences, Tehran, Iran; 4 Department of Mathematics, Islamshahr Branch, Islamic Azad University, Islamshahr, Iran; 5 Faculty of Health Sciences, Ege University, Izmir, 35575 Turkey; 6 Nursing and Midwifery Care Research Centre, School of Nursing and Midwifery, Tehran University of Medical Sciences, Tehran, Iran; 7 Social Determinants of Health Research Center, Research Institute for Health Development, Kurdistan University of Medical Sciences, Sanandaj, Iran; 8 Social Determinants of Health Research Center, Birjand University of Medical Sciences, Birjand, Iran; 9 Unit of Obstetrics and Gynecology, “Paolo Giaccone” Hospital, Department of Health Promotion, Mother and Child Care, Internal Medicine and Medical Specialties (PROMISE), University of Palermo, Palermo, Italy; 10 Department of Public Health, North Dakota State University, Fargo, ND 58108 USA; 11 Department of Computer Engineering, Amirkabir University of Technology (AUT), Tehran, Iran

**Keywords:** Ovarian cancer, Convolutional neural network, Diagnostic accuracy

## Abstract

**Background:**

Accurate detection of ovarian cancer is crucial for effective treatment and patient survival.

**Objectives:**

This study aims to evaluate the diagnostic performance of convolutional neural network (CNN) algorithms for the identification of ovarian cancer.

**Search strategy:**

In this systematic review with meta-analysis, we conducted a comprehensive literature search across four electronic databases: Medline (PubMed), Scopus, Embase, and Web of Science (WOS) in June 2024 and was subsequently updated on 1 February 2026. The search strategy was developed in consultation with domain experts and information specialists to maximize both sensitivity (SE) and specificity (SP). A combination of Medical Subject Headings (MeSH) and free-text terms related to “ovarian cancer,” “convolutional neural networks,” “deep learning,” and “artificial intelligence” was used, with Boolean operators (“AND,” “OR”) applied to combine search terms effectively.

**Selection criteria:**

Our review included all observational studies evaluating CNN algorithms for ovarian cancer detection, regardless of geographical location. Study selection was managed using EndNote and involved a two-step screening process, with titles/abstracts and full texts independently assessed by reviewers. Studies reporting the diagnostic performance of CNN algorithms for histopathologically confirmed ovarian cancer were eligible for inclusion. For the meta-analysis, we included studies that provided extractable data on true positives, false positives, true negatives, and false negatives, or threshold-specific SE and SP that could be converted into a 2 × 2 format.

**Data collection and analysis:**

Data were analyzed using R (version 4.2.3). Pooled SE, SP, and Area Under the Curve (AUC) were calculated using a multilevel hierarchical model with a study-level random effect. Four subgroups—imaging modalities, CNN architectures, learning algorithms, and database types—were investigated. Meta-regression was performed, and potential publication bias was assessed using Deeks’ funnel plot of log(DOR) versus 1/Effective Sample Size.

**Results:**

Following a review of 1,043 publications on CNN algorithms for ovarian cancer detection, 47 studies were included in the systematic review and 20 in the meta-analysis. Pooled analysis showed that CNN algorithms achieved a SE of 0.94 (95% CI 0.92–0.96), SP of 0.95 (95% CI 0.90–0.97), and an AUC of 0.974 (95% CI 0.961–0.981). Among imaging modalities, magnetic resonance imaging (MRI) demonstrated the highest diagnostic performance (SE 0.97, SP 0.955, AUC 0.986), followed by computed tomography (CT) (SE 0.914, SP 0.975, AUC 0.983), histopathology (SE 0.979, SP 0.934, AUC 0.981), and ultrasound (SE 0.891, SP 0.951, AUC 0.922). Among CNN architectures, other architectures achieved the highest pooled AUC (0.979), followed by ResNet (SE 0.92, SP 0.947, AUC 0.969) and DenseNet (SE 0.927, SP 0.932, AUC 0.956). Transfer learning (SE 0.942, SP 0.948, AUC 0.978) outperformed fully trained models (SE 0.959, SP 0.929, AUC 0.962). Open-source datasets showed higher performance (SE 0.98, SP 0.971, AUC 0.985) than non-open datasets (SE 0.931, SP 0.938, AUC 0.968). Meta-regression indicated that the “other” algorithm family was significantly associated with higher logDOR, while imaging modality, dataset openness, transfer learning, and DenseNet were not significant predictors. Substantial heterogeneity remained across studies, but leave-one-out analysis confirmed the robustness of the pooled estimates, and Deeks’ test suggested potential publication bias.

**Conclusion:**

CNN-based algorithms demonstrate high diagnostic accuracy for ovarian cancer detection, with particularly strong performance across imaging modalities such as MRI, CT, and histopathology. These findings highlight the potential of deep learning models to support AI-assisted diagnostic workflows and improve early detection. However, substantial heterogeneity across studies and potential publication bias indicate the need for standardized imaging protocols, larger multi-center datasets, and external validation. Future research should focus on harmonizing data sources and integrating CNN-based tools into clinical decision-making to enhance diagnostic reliability and patient outcomes.

**Supplementary Information:**

The online version contains supplementary material available at 10.1186/s12911-026-03462-9.

## Introduction

Ovarian cancer is a critical global health issue, characterized by high mortality rates and the need for accurate diagnostic tools [1]. Timely and precise diagnosis is essential for improving patient outcomes and guiding treatment strategies. Medical imaging techniques, including magnetic resonance imaging (MRI), ultrasound (US), computed tomography (CT), and positron emission tomography (PET), are commonly used for diagnosing and staging ovarian cancer; [2]; however, their accuracy is often limited, highlighting the need for more effective diagnostic approaches [3, 4]. Recently, deep learning (DL), particularly convolutional neural networks (CNNs), has shown great promise in enhancing the accuracy of ovarian cancer diagnosis [5, 6]. CNNs can automatically extract complex features from medical images and generally outperform traditional machine-learning and diagnostic methods [7, 8] especially when employing advanced architectures such as residual networks (ResNet) and densely connected networks (DenseNet) [9]. Unlike conventional histopathological examination, which is time-consuming and dependent on specialist interpretation, CNN-based analysis is non-invasive, objective, and rapid [10–13]. Moreover, the performance of CNNs can be further enhanced using transfer learning, which leverages pretrained models to improve accuracy, especially for limited or heterogeneous datasets [14, 15]. Beyond model optimization techniques such as transfer learning, the diagnostic accuracy of CNNs also depends on the type of medical imaging modality. Histopathological images typically yield the highest performance due to their high-resolution cellular details, while MRI, CT, and US images can be improved through advanced preprocessing and domain-specific CNN architectures [16, 17]. Supporting these advantages, multiple studies have demonstrated that CNNs can classify ovarian tissue with high accuracy, often comparable to experienced radiologists, and have also shown potential generalizability across various cancer types [18–27]. The integration of DL into clinical practice holds significant potential to improve patient outcomes through more accurate diagnosis and treatment planning [28]. his meta-analysis aims to evaluate the diagnostic accuracy of CNN algorithms in detecting ovarian cancer across different medical imaging modalities.

## Materials and methods

### Protocol and guideline

This systematic review and meta-analysis was conducted according to the Preferred Reporting Items for Systematic Reviews and Meta-Analyses (PRISMA) guidelines and was registered in the International Prospective Register of Systematic Reviews (PROSPERO ID: CRD42024552290). Formal ethical approval was not required. Initially registered on PROSPERO as a systematic review on 29 May 2024, the protocol was updated on 5 August 2024 once a meta-analysis became feasible. All planned subgroup and meta-regression analyses were pre-specified in the revised registration.

The main goal of this study is to assess the diagnostic accuracy of CNN applied to medical imaging techniques for detecting ovarian cancer. The PICO question for this analysis is as follows: P – Population: Patients with ovarian cancer (according to the ICD-10 code C56.9, confirmed by histology); C – Comparison: CNN algorithms versus pathology reports as the gold standard; O – Outcome: diagnostic accuracy. In this study, medical imaging techniques included ultrasound (abdominal, transvaginal, and color Doppler), CT, MRI, [18 F] FDG PET/CT, and histopathological images.

### Search strategy and data sources

We conducted an extensive search for relevant articles across three reputable databases: PubMed/MEDLINE, Scopus, and Web of Science. The literature search was initially conducted in June 2024 with no restrictions on publication date or language and was subsequently updated on 1 February 2026. A comprehensive search was performed in PubMed using the following search strategy: ((Computer Neural Network[Title/Abstract]) OR (Computer Neural Networks[Title/Abstract])) OR (Network Model, Neural[Title/Abstract])) OR (Neural Network Model[Title/Abstract])) OR (Computational Neural Networks[Title/Abstract])) OR (Computational Neural Network[Title/Abstract])) OR (Neural Networks, Computational[Title/Abstract])) OR (Perceptrons[Title/Abstract])) OR (Perceptron[Title/Abstract])) OR (Connectionist Models[Title/Abstract])) OR (Connectionist Model[Title/Abstract])) OR (Model, Connectionist[Title/Abstract])) OR (Neural Networks (Computer[Title/Abstract]))) OR (Neural Network (Computer[Title/Abstract]))) OR (“Neural Networks, Computer“[Mesh])) AND ((“Ovarian Neoplasms“[Mesh]) OR ((((((((((Neoplasm, Ovarian[Title/Abstract]) OR (Ovarian Neoplasm[Title/Abstract])) OR (Ovary Neoplasms[Title/Abstract])) OR (Ovary Neoplasms[Title/Abstract])) OR (Neoplasms, Ovarian[Title/Abstract])) OR (Ovary Cancer[Title/Abstract])) OR (Ovary Cancer[Title/Abstract])) OR (Ovarian Cancer[Title/Abstract])) OR (Cancer of Ovary[Title/Abstract])) OR (Cancer of Ovary[Title/Abstract]))) Sort by: Most Recent. To refine the search, we applied MeSH keywords and Boolean operators (such as AND, OR). One reviewer (L.A) manually reviewed the reference lists of relevant papers.

### Study selection and eligibility criteria

The study selection process was managed using EndNote software (version X9, Thomson Reuters), which helped list and screen studies. After removing duplicate articles, eligibility screening was carried out in two steps. First, two trained authors (L.A and A.KA) evaluated articles by title and abstract to assess their relevance to the research topic. A total of 143 articles were identified as potentially relevant and moved on to the full-text review. During this phase, two authors (L.A and S.H) independently assessed the full-text articles against the inclusion criteria using a checklist-style form.

We included studies that reported the diagnostic performance of CNN models for detecting ovarian cancer using medical imaging alone, including radiologic modalities (ultrasound, CT, MRI) and histopathological images, with histopathology serving as the reference standard where applicable. We excluded non-English publications, reviews, editorials, letters, authors’ replies, conference abstracts, animal studies, preprint manuscripts, and articles unavailable in full text. Studies in which image segmentation or lesion localization constituted the primary objective or final output of the model were excluded. Furthermore, studies were excluded if they incorporated clinical variables (e.g., age, menopausal status, CA-125, or other serum biomarkers) as inputs to the diagnostic classifier. In addition, studies focusing on ovarian cyst differentiation or other gynecologic or non-gynecologic malignancies were excluded. Finally, studies aimed at predicting metastasis, recurrence, prognosis, treatment response, patient monitoring, or personalized therapeutic outcomes, rather than primary diagnostic classification, were not considered eligible. Research related to gene expression profiling, immunohistochemistry (protein markers), nuclear morphometric features, nuclear lamin protein distribution, or serum biomarker analysis was excluded. Moreover, studies using non-conventional light microscopy modalities, such as fluorescence imaging, hyperspectral imaging, or second-harmonic generation, were excluded if they did not align with the study objectives. Studies relying solely on human-assisted image classification without an independent CNN-based diagnostic output were excluded.

**Fig. 1:**
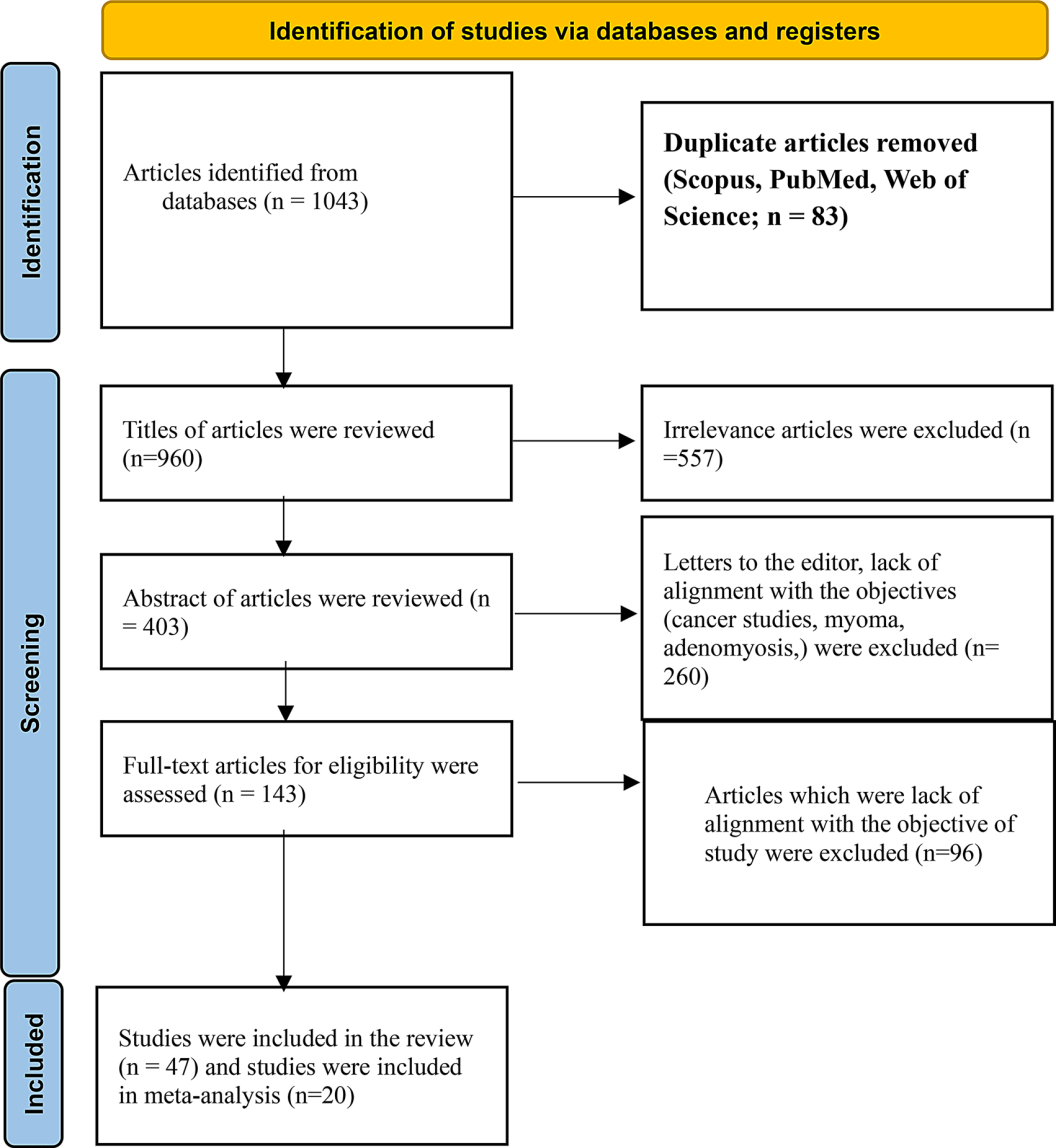
PRISMA Flowchart of search process and results

To clarify our selection process, we used a PRISMA flowchart (Fig. 1) to visually illustrate how studies were screened and included in our review.

### Outcomes

The primary objective of this study was to comprehensively evaluate the diagnostic performance of CNNs when applied to various medical imaging modalities for the detection of ovarian cancer. Specifically, we aimed to quantify key performance metrics such as sensitivity (SE), specificity (SP), accuracy, precision (positive predictive value (PPV), negative predictive value (NPV), F-score, and the area under the receiver operating characteristic curve (AUC). Additionally, the study sought to synthesize evidence across different imaging modalities and CNN architectures to identify factors influencing diagnostic accuracy and to assess the generalizability of these algorithms across diverse clinical settings.

### Data synthesis and extraction

The two reviewers independently collected data from the selected studies using a custom data extraction table in Microsoft Excel. All relevant studies were included in the review. Key information was systematically collected to support comprehensive analysis and comparison, including study details (title, first author, and publication year), participant age (mean or median, with standard deviation or range), number of participants, source of data, open-access dataset availability, types of medical imaging modalities, total number of images, number of images for training, validation, internal, and external sets, type of internal validation, external validation, exclusion of poor-quality imaging, use of transfer learning, CNN architecture, sample characteristics, image pixel information, measured outcomes, and diagnostic performance metrics including SE, SP, PPV, NPV, F1-score, accuracy, and AUC. Discrepancies between reviewers were resolved through discussion, with input from a third expert when necessary (MM.GH). Only studies providing extractable 2 × 2 data or threshold-specific SE and SP convertible into 2 × 2 tables were included in the meta-analysis.Binary diagnostic accuracy data were entered into contingency tables with true positives, false positives, true negatives, and false negatives to calculate pooled SE, SP, and other metrics. When studies reported multiple contingency tables for the same or different CNN architectures, each table was treated as independent.

### Assessment of risk bias

The risk of bias in individual studies was evaluated using the Quality Assessment of Diagnostic Accuracy Studies-2 (QUADAS-2) checklist [29]. Each article was independently evaluated by the reviewers using these criteria, and disagreements were resolved by discussion.

### Statistical analysis

Statistical analyses were conducted using R (version 4.2.3) with the packages “dplyr,” “metafor,” “mada,” and “ggplot2,” as well as the robvis risk-of-bias. Hierarchical summary receiver operating characteristic (HSROC) modeling was used to evaluate the diagnostic performance of the CNN algorithms, including pooled SE, SP, and AUC with corresponding 95% confidence intervals. To appropriately handle multiple 2 × 2 contingency tables originating from the same study, a bivariate HSROC model with a study-level random effect was applied. This multilevel specification prevents double counting and accounts for within-study correlation. After this correction, each study contributed either a single representative table or an aggregated set of tables. Zero cells in the 2 × 2 tables—which can lead to instability in logit-based estimation—were addressed by applying a continuity correction of 0.5 only when required by the model implementation, and all corrected cells are reported for transparency [30]. A threshold-effect assessment was conducted using the Spearman correlation between logit (SE) and logit(1–SP). The HSROC figures display the summary curve together with the corresponding 95% confidence regions and the prediction region produced by the bivariate model. Prediction intervals for SE, SP, as well as the variance components (τ²) on the logit scale, were also estimated. We also performed subgroup meta-analyses and regression analyses to investigate potential sources of heterogeneity. Subgroup meta-analyses were performed based on the following criteria: (1) imaging modalities, with group 1 using histopathological images, group 2 using ultrasound images (abdominal, vaginal, color Doppler), group 3 using CT/[18 F]FDG PET/CT images, and group 4 using MRI images; (2) CNN algorithms, with group 1 using ResNet, group 2 using DenseNet, and group 3 using other CNN algorithms (AlexNet, DCNN, DFCNN, GoogLeNet, Hybrid (CNN & GoogLeNet V3), Hybrid (Inception-ResNet v2), Hybrid CNN–Transformer model, InceptionV3, NASNetLarge, NASNetMobile, OVANet (Ensemble CNN: VGG19 + InceptionV3 + SE + spatial attention), OvCan-FIND, MobileNet, VGG16, VGG19, Xception, Xception_ViT); (3) learning type algorithms, categorized into transfer learning and full learning; and (4) type of databases, comparing open-source versus non-open-source datasets. The forest plot of the studies was generated. To assess potential publication bias, we utilized. Deeks’ funnel plot of log(DOR) against 1/Effective Sample Size. The threshold for statistical significance was set at *p* < 0.05, and all tests were two-sided.

## Result

A total of 1043 publications were initially identified from various databases. After removing 83 duplicates retrieved from Scopus, PubMed, and Web of Science, 960 unique articles remained. Titles and abstracts were screened, and eight authors were contacted to request full texts [18, 31–37] with two authors responding [18, 36]. Ultimately, 143 articles were available for full-text review. Studies on image segmentation, feature extraction, gene expression, immunohistochemistry, mixed cancer types, or non-conventional imaging (*n* = 84) were excluded. Additionally, one preprint [38], two retracted studies [39, 40], and nine studies with unusable data were excluded [41–49]. Finally, the present review included 47 studies (Fig. 1), categorized by imaging modality as follows: histopathology (*n* = 13) [18, 19, 36, 50–58], ultrasound (*n* = 14) [8, 25, 59–69], CT (*n* = 12) [20, 70–80], and MRI (*n* = 8) [23, 24, 37, 81–85]. Study characteristics are summarized in Tables 1, 2, 3, 4 and 5. None of the studies reported a prespecified sample size calculation. Twenty-one studies used random split-sample validation, and three used k-fold cross-validation. ResNet and DenseNet were the most commonly used algorithms for detecting ovarian cancer from ultrasound images. Out of the 47 studies reviewed, 20 provided sufficient data to create contingency tables for diagnostic performance and were included in the meta-analysis [18, 19, 25, 34, 36, 52, 53, 55, 57, 59, 61–63, 69, 71, 76, 78, 80, 85].

**Table 1:** Diagnostic accuracy of CNNS in detection of ovarian cancer based on histopathological images

First author and year	Age Mean or median (SD; range)	Number of participants	Type of internal validation	External validation	Exclusion of poor-quality imaging	Source of data	Open access data	Transfer/fully learning applied	Number of total images	Number of images for training/ validation/ internal/ external	Original/ Augmented	Feature classification	number of Images (training)	Algorithm architecture	Sensitivity (%)	Specificity (%)	Precision (PPV)	NPV	F1-score	Accuracy (%)	AUC
Wu et al. 2018 [50]	NR	85	10-fold cross-validation	NR	No	Hospital of Xinjiang Medical University	No	Fully learning	1848	NR/ NR/NR/NR	Original	Serous	481	DCNN (AlexNet)	NR	NR	NR	NR	NR	82.33	NR
												Mucinous	453		NR	NR	NR	NR	NR	71.62	NR
												Endometrioid	484		NR	NR	NR	NR	NR	64.53	NR
												Clear cell	430		NR	NR	NR	NR	NR	72.57	NR
Kasture et al. 2021 [19]	NR	NR	NR	NR	NR	National Cancer Institute’s Genomic Data Commons data portal, TCGA-OV repository and GDC Data Portal	Yes	Fully learning	Original images (500 samples)	NR/NR/NR/NR	Original	Serous	175	CNN (AlexNet)	0.88	NR	0.92	NR	0.92	75	NR
												Mucinous	100		0.87	NR	0.75	NR	0.87	70	NR
												Endometroid	60		0.89	NR	0.9	NR	0.9	79	NR
												Clear Cell	80		0.9	NR	0.85	NR	0.89	70	NR
												Non-Cancerous	85		0.8	NR	0.85	NR	0.83	71	NR
Wadhwa et al. 2021 [51]	NR	48	NR	NR	Yes	PLCO dataset	Yes	NR	NR	NR/NR/NR/NR				DenseNet-201	98.9	NR	91	NR	95	94.73	92.9
Ramasamy & Kaliyaperumal, 2023 [18]	NR	NR	NR	NR	NR	TCGA-OV.	Yes	Fully learning	776	582/NR/194/NR	NR	Classification of ovarian cancer stage	NR	DFCNN	99.65,	98.42	99.21	NR	NR	99.22	0.99
Sundari & Brintha, 2023 [36]	NR	NR	NR	NR	NR	National Cancer Institute Genomic Data Commons Data Portal	Yes	Transfer learning	18,000	14,400/NR/3600/NR	Original	Serous	570	ResNet	96.08	97.02	97.56	NR	96.72	97.82	0.99
												Mucinous	358								
												Endometrioid	312								
												Non-cancerous	220								
												Clear cell	340								
Ziyambe et al. 2023 [52]	NR	NR	Random split-sample validation	NR	NR	Cancer Genome Atlas TCGA repository	Yes	Transfer learning	11,040	8832/NR/2208/NR		serous ovarian cancer and non-cancerous samples		Hybrid (CNN & GoogleNet (V3))	0.9502	0.9316	0.9302	NR	0.94	94.43	NR
Kasture et al. 2024 [53]	NR	NR	Random split-sample validation	NR	No	Cancer Repository	Yes	Fully learning	25,742	24,742.NR/1000/NR		Subtype Classification of Ovarian Cancer		AlexNet	NR	NR	NE	NR	NE	70%	NE
														VGG-19	NR	NR	90%	NR	NE	90%	NE
Asadi-Aghbolaghi, et al. 2024 [54]	NR	523 (source), 60 (target)	3-fold cross-validation	Target domain evaluation	NR	Source and target WSIs from two centers	No	Adversarial training with FFT-Enhancer, SVM aggregation	1053 WSIs (source), 60 WSIs (target)	150 patches per slide; 1024 × 1024 pixels resized to 512 × 512	Original	High-grade serous carcinoma	446	ResNet18 + FFT-Enhancer + VLAD + SVM	NR	NR	NR	NR	NR	75.82 (target), 80.68 (source)	NR
													101		NR	NR	NR	NR	NR	NR	NR
												Endometrioid	257		NR	NR	NR	NR	NR	NR	NR
												Clear Cell	173		NR	NR	NR	NR	NR	NR	NR
												Low grade serous carcinoma	76		NR	NR	NR	NR	NR	NR	NR
Fahim et al. 2024 [55]	NR	NR	N5-fold cross-validation + HoldoutR	No	NR	SDM College of Medical Sciences and Hospital, Dharwad, Karnataka, India	Yes	Transfer learning	508 (original)	407 / NR / 101 / NR	Original	Clear cell	83	OVANet (Ensemble CNN: VGG19 + InceptionV3 + SE + spatial attention)	100	100	1	1	1	0. 985	0. 985
												Endometrioid	85		100	100	1	1	1		
												Mucinous	77		100	98.77	0.95	1	0.97		
												Serous	85		100	100	1	1	1		
												Non-cancerous	77		95	100	1	0.98	0.97		
Sundari & Brintha, 2024 [34]	NR	NR	80:20 train-test split	NR	NR	National Cancer Institute – Genomic Data Commons (GDC) Data Portal	Yes	Transfer learning with pretrained ResNet-50	18,000 (after augmentation)	Training: 14,400 images (80%) Testing: 3,600 images (20%)	Original:	Serous	570	ResNet-50	96.08	97.02	97.56	NR	96.72	98.82	99
												Mucinous	358								
												Endometrioid	312								
												Non-cancerous	220								
												Clear cell	340								
Bikku et al. 2025 [56]	NR	NR	Hold-out validation	No	NR	Public datasets (IEEE DataPort – STRAMPN; Mendeley Data)	Yes	Transfer learning (DenseNet121, ResNet50, VGG16/19, MobileNet)	1485	Training: 1198 (729 cancer, 469 non-cancer); Hold-out test: 299 (157 cancer, 142 non-cancer); External: 0	Orginal	Binary classification (Cancerous vs. Non-cancerous)	Final dataset after augmentation: 6263	DenseNet121	93	92	94	91	93	NR	NR
														ResNet50	94	90	93	92	94	NR	NR
														VGG16,	91	92	92	90	91	NR	NR
														VGG19	90	91	91	89	91	NR	NR
														MobileNet;	89	87	90	88	89	NR	NR
Saha et al. 2025 [57]	NR	42	Split train/val/test (80/10/10)	NR	NR	Smt. Kashibai Navale Medical College & The Cancer Repository	Yes (Cancer Repository)	Fully transfer learning	85	Train: 68, Validation: 8, Test: 9	Augmented	Histopathology images of OC subtypes	68	OvCan-FIND	99.56	99.68	99.68	NR	99	99.74	NR
Lee et al. 2025 [58]	NR	NR	Random split (8:1:1) into training / validation / test sets at WSI level	Yes (independent external dataset from Seoul National University Bundang Hospital)	Yes	OPEN AI dataset (Korean National Information Society Agency, AI-Hub)	Yes	Transfer learning applied	Total patch images (PIs): 22,724	Patch images (PIs): 14,699 benign 8,025 ovarian cancer External validation: 131 WSIs	Augmented	(Benign vs. Ovarian cancer)	NR	ResNet50 (CNN, fully supervised, patch-based)	0.86	0.61	NR	NR	NR	0.779	0.80

**Table 2:** Diagnostic accuracy of CNNS in detection of ovarian cancer based on ultrasound images (US)

First author and year	Age Mean or median (SD; range)	Number of participants	Source of data	Open access data	Type of internal validation	External validation	Exclusion of poor-quality imaging	Total image	Modality	Number of images for training/ validation/ internal/ external	Transfer/ fully learning	Algorithm architecture	Feature classification	Sensitivity	Specificity	Precision (PPV)	NPV	F1-score	Accuracy	AUC	
Wang et al. 2021 [59]	NR	265	Tianjin Medical University Cancer Institute and Hospital	No	Random split-sample validation	No	NR	279	US	195/أNR/84/NR	Transfer Learning	VGG16	Normal vs. Borderline & malignant	0.931	0.771	NR	NR	0.843	0.871	0.897	
												GoogLeNet		0.828	0.972	NR	NR	0.894	0.883 (± 0.019)	0.924	
												ResNet34		0.914	0.914	NR	NR	0.914	0.914	0.963	
												MobileNet		0.931	0.771	NR	NR	0.843	0.871	0.885	
												DenseNet		0.983	0.686	NR	NR	0.808	0.871	0.877	
											Fully learning	VGG16		0.931	0.686	NR	NR	0.790	0.839	0.886	
												GoogLeNet		0.845	0.917	NR	NR	0.880	0.872	0.914	
												ResNet34		0.966	0.771	NR	NR	0.858	0.893	0.909	
												MobileNet		0.948	0.686	NR	NR	0.796	0.850	0.870	
												DenseNet		0.966	0.657	NR	NR	0.782	0.850	0.900	
Chen et al. 2022 [25]	mean age, 46.4 years 6 14.8	422	Hospital dataset	No	Random split-sample validation	NR	Yes	2113	multimodal US (gray scale and color Doppler US images)	1493/189/431/NR	Transfer learning	ResNet	malignant from benign ovarian tumors	92	80	65	96	NR	NR	0.9	
Gao et al. 2022 [8]	training dataset (32 (27–42), internal validation dataset (38 (27–49), external validation dataset 1 (43 (32–52),external validation dataset 2 (38 (27–48)	107,624	Ten hospitals across China	No	Random split-sample validation	Yes (Two External validation)	Yes	592,272	US	575 930/NR/868/2092	Transfer learning	DenseNet-121	malignant from benign ovarian tumors	78·9%	93·2%	83·7%	90·9%	0·812	88·8%	0·911	
Jung et al. 2022 [7]	NR	1154	Seoul St. Mary’s Hospita	No	5-fold cross-validation	NR	NR	1613	US	NR/NR/1613/NR	Transfer	DenseNet121	Normal from other malignant tumors	97.22	97.21	84.28	99.56 (99.19– 99.94)	NR	97.22 (96.68– 97.76)	0.9936 (0.9914– 0.9958)	
												DenseNet161		90.70	98.29	89.1	98.57	NR	97.28	0.9918	
Alwan et al. 2023 [60]	NR	196	Zhejiang University’s School of Medicine’s Affiliated Women’s Hospital	No	Random split-sample validation	NR	NR	4090	US	2454/NR/1636/NR	Transfer learning	ConvNet	Benign and malignant tumor	NR	NR	NR	NR	NR	98.79	NR	
Miao et al. 2023 [61]	The age of the patients was 42.09 ± 13.17, 41.31 ± 16.53, and 43.51 ± 13.73 years in the TVS set, TAS set, and CDFI_TVS set	1350	Fourth Affiliated Hospital of Harbin Medical University	No	Random split-sample validation	No	Yes	3287	TVS	1304 train/326 test	Transfer learning	ResNet	Classifying malignant and benign tumors	97	93	92	97	NR	NR	0.95	
									TAS	622 train/154 test				95	95	95	95	NR	NR	0.95	
									(CDFI_TVS)	705 train/176 test				83	94	92	86	NR	NR	0.88 [0.84–0.93]	
Wu et al. 2023 [62]	NR	328	Beijing Shijitan Hospital	No	Random split-sample validation	NR	Yes	1142	US	799/114/228/NR	Transfer learning	VGG16	Benign and malignant tumor	0.679	0.958	0.715	0.95	0.693	0.95	0.768	
												GoogleNet		0.563	0.943	0.607	0.94	0.571	0.87	0.671	
												ResNet34		0.695	0.961	0.64	0.95	0.662	0.95	0.763	
												ResNext50		70.2	96.7	0.708	0.96	0.7	0.95	0.811	
												DensNet121		0.576	0.947	0.58	0.94	0.578	0.92	0.693	
												DensNet201		0.702	0.96	0.73	0.95	0.711	0.96	0.768	
Xi et al. 2023 [63]	benign group [55 years (IQR 49–64) vs. 35 years (30–45) in training set; 56 years (IQR 49–66) vs. 33 years (28–44) in the validation set].	405	Hospital of Soochow University	No	Random split-sample validation	No	Yes	1103	US	772/NR/331/NR	Fully learning	MobileNet	Differentiate of malignant and benign tumors	0.747	0.795	0.741	0.799	0.744	0.773	0.836	
												Xception		0.863	0.941		0.920	0.89	0.906	0.968	
												Inception		0.973	0.849		0.835	0.975	0.903	0,987	
												ResNet		0.945	0.957	0.945	0.957	0.945	0.952	0.988	
												DenseNet		0.952	0.973	0.965	0.963	0.969	0.964	0.997	
Giourga, et al. [64]	-	585	Single-center tertiary gynecological oncology center	No	k-fold cross-validation	No	Yes (manual + auto ROI cropping)	3510	US	3510 (6 images per patient; training/validation/internal splits per k-fold)	Transfer learning	VGG16	Differentiate of malignant and benign tumors	95.50	83.60				87.50	89.50	
												ResNet50,		90.20	84.90				86.80	87.50	
												InceptionNet		88.70	88.90				88.90	88.70	
												aggregate weighted		96.50	88.10				90.90	92.20	
Kongara et al. 2024 [65]	NR	NR	Public ovarian cyst ultrasound dataset by Zhang et al.; additional images from Peking Union Medical College Hospital	Partially	fixed train/validation/test split (806 / 420 / 394)	No	NR	1,620 ultrasound images	US	Training: 806; Validation: 420; Testing: 394 (derived from total); External: nr	Fully supervised CNN	KHO-CNN (custom convolutional neural network architecture)	Benign	86.49	NR	87.11	NR	86.36	85.78	NR	
													malignant	87.51	NR	86.4	NR	86.04	85.79	NR	
Pham and Le 2024 [66]	NA	NA	Multi-modality ovarian tumor ultrasound	No	Fine-tuning YOLOv7 & YOLOv8	NA	Yes	1469 (2D US images)	US	Training: 1000, Validation/Internal: 469, External: —	Transfer learning (fine-tuning)	YOLOv8 (n, s,m, l,x), YOLOv7 variants	Ovarian tumor detection	68.7	NA	71.5	NA	NA	66.8	NA	
Wang et al. 2024 [67]	40.8–42.4 (range)	1054	Shenzhen People’s Hospital	No	5-fold cross-validation	Yes	Yes	4542	US	Training: 675, Validation: 169, Testing: 210	transfer learning from ImageNet	ResNet-50	Benign vs. malignant	94.28	87.62	NA	NA	NA	90.00	0.957	
Wang et al. 2025 [68]	44–46 (mean ± SD: 44 ± 15, 45 ± 15, 46 ± 14)	997 patients	5 hospitals in China (3 for training/validation, 2 for external test)	No	5-fold cross-validation (training/validation 4:1 split)	Yes (2 hospitals)	Yes	1417	US	Training 1055 / Validation 264 / Test 98	Transfer learning (pretrained ImageNet weights)	ResNet-50 (DL visual model) + LLM integration	Benign / Borderline / Malignant	85–95	76–95	63–95	71–95	76–95	85–95	NR	
Garcia-Atutxa et al. 2025 [69]	NR	NR	OTU-2D ultrasound dataset (public ovarian tumor ultrasound dataset)	Yes	5-fold cross-validation	NR	NR	1,146 ultrasound images	US	NR	Transfer learning applied	Hybrid CNN–Transformer model (ResNet / DenseNet / EfficientNet backbones + Swin Transformer)	Multiclass classification	0.93	0.99			0.93	0.93	0.99	

**Table 3:** Diagnostic accuracy of CNNS in detection of ovarian cancer based on CT images

First author and year	Age Mean or median (SD; range)	Number of participants	Source of data	Open access data	Type of internal validation	External validation	Exclusion of poor-quality imaging	Transfer/fully learning applied and number of images		Number of images for training/ validation/ internal/ external	Feature classification	Algorithm architecture	Sensitivity	Specificity	Precision (PPV)	NPV	F1-score	Accuracy	AUC
Nagarajan &Tajunisha, 2021 [70]	NR	NR	TCGA-OV) dataset	Yes	NR	NR	NR	NR	497	350/NR/147/NR		DCNN-AlexNet	NR	NR	NR	NR	NR	87.84%	NR
Arathi & Shanthini, 2022 [20]	NR	NR	(TCGA-OV)	Yes	Random split-sample validation	NR	NR	Transfer learning	141	71/NR/NR/NR		Efficient Net	98.8	99.8	NR	NR	NR	99.8	NR
Kodipalli et al. 2022 [71]	NR	NR	Shri Kshetra Dharmasthala (SDM) College of Medical Sciences and Hospital located at Manjushreenagar in Dharwad, Karnataka, India, and Kempegowda Institute of Medical Sciences., Karnataka, India	No	Random split-sample validation	NR	NR	Transfer learning	5205	3644/1561/510/NR	benign and malignant	Inception-ResNet v2	84	90	74	NR	79	67	NR
Boyanapalli & Shanthini, 2023 [72]	NR	NR	TCGA-OVdataset	Yes	Random split-sample validation	NR	NR	Transfer learning	NR	NR/NR/NR/NR	classify the different types of OC	ResNet, VGG-16, LeNet)-IAO	94.234	92.61	93.32	NR	92.59	96.53	NR
Kodipalli et al. 2023 [74]	NR	NR	SDM Dharwad College and Hospital	No	NR	NR	NR	Transfer learning	20	NR/NR/NR/NR	benign and malignant	CNN	88	NR	89.1	NR	89.40	89.7	NR
												ResNet 152	91.50	NR	92.40	NR	92.70	92.70	NR
												DenseNet121	94.30	NR	95.2	NR	95.6	95.7	NR
												Inception-ResNet V4	93.20	NR	94.1	NR	94.2	94.3	NR
												VGG 16	90.5	NR	91.6	NR	91.4	91.5	NR
												Xception	86.7	NR	87.3	NR	86.3	87.2	NR
Kodipalli et al. 2023 [73]	NR	53	SDM College of Medical Sciences and Hospital, Dharwad, India	No	Random split-sample validation	NR	NR	Transfer learning	5725	4164/NR/1561/NR	benign and malignant	ResNet60	NR	NR	NR	NR	NR	97.5	NR
Nagarajan &Tajunisha, 2023 [75]	NR	NR	TCGA-OV dataset	Yes	NR	NR	NR	NR	497	350/ NR/147/NR	classifying the ovarian tumor types	DCNN	NE	NR	NE	NR	NE	93.82	NR
Sadeghi et al. 2023 [76]	mean age 56.3 years; age range 36–83 years	37	Kowsar Hospital	No	Random split-sample validation	NR	NR	Fully learning	1224	1054/NR/170.NR	classification (cancerous vs. noncancerous)	3D CNN based on ResNet-50	99%	NR	88%	NR	93	92	0.99
											Staging (III–IV)		95	NR	92	NR	94	0.94	0.990
Kodipalli, et al. 2024 [77]	NR	349	SDM Medical College and Science, Dharwad	No	Random split-sample validation	NR	NR	Transfer learning	NR	NR/NR/NR/NR		ensamble (VGG16, ResNet 152, Inception V3, and DenseNet 101)	NE	NR	97.44	NR	98.7	98.96	NR
Guha et al. 2024 [78]	NR	53	SDM College of Medical Sciences, India	No	Hold-out split (train/validation/test)	No	Yes (annotation-based cropping)	Transfer learning images total	5725	Train: 3644 / Validation: 1561 / Test: 520 / External: 0	Benign vs. Malignant	Enhanced ResNet50 (ResNet60)	100	90.97	96.66	NR	98.3	97.5	NR
Li et al. 2025 [79]	NR	868 total (463 ovarian tumor patients + 405 healthy individuals)	Private CT dataset from local medical institutions	No	Train/validation/test split (7:2:1 ratio)	Yes; 53 patients from a separate hospital (34 benign, 19 malignant)	Yes (8 patients excluded due to poor imaging quality)	Fully supervised learning with supervised contrastive learning	NR	Total CT images: 2,512 (1,485 normal, 540 benign, 487 malignant); detailed split numbers per set: NR	Benign vs. Malignant	CNN with attention and supervised contrastive learning	98.43			98.42	98.41	98.43	
Alshdaifat et al. 2025 [80]	NR	500	King Abdullah University Hospital (KAUH), Jordan	No	NR	No	NR	Transfer learning	NR	NR	Benign vs. Malignant	Xception_ViT	95.96	99.22	96.14	95.88	NR	96.05	NR

**Table 4:** Diagnostic accuracy of CNNS in detection of ovarian cancer based on MRI images

First author and year	Age Mean or median (SD; range)	Number of participants	Type of internal validation	External validation	Exclusion of poor-quality imaging	Source of data	Open access data	Transfer/fully learning applied	Total images	Number of images for training/ validation/ internal/ external	Algorithm architecture	Feature classification	Sensitivity	Specificity	Precision (PPV)	NPV	F1-score	Accuracy	AUC
Shafi & Sharma, 2019 [81]	NR	NR	NR	NR	NR	skims (sher-i-kashmir institute of medical science) and Hospital Kashmir.	No	NR	250	NR/NR/NR/NR/NR	ABC-CNN	Normal (50)	0.90	0.91	NR	NR	NR	0.98	NR
												Stage 1 (50)	0.92	0.93	NR	NR	NR	0.98	NR
												Stage 2 (50)	0.95	0.96	NR	NR	NR	0.98	NR
												Stage 3 (50)	0.98	0.97	NR	NR	NR	0.99	NR
												Stage 4 (50)	0.99	0.98	NR	NR	NR	0.99	NR
Wang et al. 2021 [82]	45.7 ± 16.7 (Benign), 49.9 ± 18.1 (Malignant)	451	4 fold ofthe cross-validation	NR	NR	Penn databases	No	NR	545	384/108/53/NR	ResNet	benign frommalignant ovarian	0.62	0.89	NR	NR	0.67	0.81	0.75
											EfficientNet		0.75	0.92	NR	NR	0.77	0.87	0.81
Jian et al. 2022 [23]	mean age, 48.93 14.05 years	501	Random split-sample validation	NR	Yes	Eight hospitals	No	NR	501	342/NR/159.NR	MICNN	Borderline and Malignant Epithelial Ovarian Tumors	0.74	0.78	NR	NR	0.81	0.76	0.884
Saida et al. 2022 [24]	mean age, 50 years; age range, 20–90 years	465	Random split-sample validation	NR	NR	Hospital	No	Transfer learning	3763	3663/NR/100/NR	Xception	ovarian carcinomas and borderline tumors	0.85	0.77	NR	NR	NR	0.81	0.89
Akazawa & Hashimoto, 2023 [83]	51 (14–84)	185	Random split-sample validation	No	Yes	Tokyo Women’s Medical University Adachi Medical Center	No	Transfer learning	NR	NR/NR/NR/NR/NR	VGG16	Benign vs. borederline and malignant tomurs	Using sagittal images, the model achieved a recall of 0.89 (95%CI = 0.85–0.92)	NR	Using sagittal images, the model achieved a precision of 0.65 (95%CI = 0.630.67)	NR	Using sagittal images, the model achieved a F1 score of 0.75 (95%CI = 0.72–0.77)	0.628 (95%CI = 0.592–0.662) using sagittal images, and 0.500 (95%CI = 0.486–0.512) using horizontal images.	Using sagittal images, the AUC of the binary classification was 0.529 (95%CI = 0.500-0.557)
Wang et al. 2023 [84]	50.3 ± 14.0 years	158 patients	Five-fold cross-validation (patient-level split)	No	Yes	National Clinical Research Center for Cancer, Chinese Academy of Medical Sciences and Peking Union Medical College	No	Fully learning	1803 MRI lesion slices	31–34 lesions 349–373 slices ~ 1312–1648 patches External: None	ResNet-18	Benign vs. Malignant ovarian tumors	0.860	0.783	NR	NR	0.817	0.824	0.916
Zheng et al. 2024 [21]	Ovarian thecoma-fibroma(59.91 ± 11.72) & Solid ovarian cancer 57.03 ± 11.82	180	Random split-sample validation	NR	NR	Hospital	NR	Transfer learning	NR	NR/NR/NR/NR/NR	ResNet18		0.848	NR	0.828	NR	NR	0.852	0.919
Amin et al. 2025 [85]	NR	350	Hold-out validation (stratified split: 80% training / 10% validation / 10% test)	No	Yes (irrelevant/duplicate slides excluded by clinicians)	King Abdullah University Hospital (KAUH), Jordan	No	Transfer learning (DenseNet121 pretrained; 93 layers frozen)	918 images	Training ≈ 734 / Validation ≈ 92 / Test ≈ 92 / External: 0	DenseNet121 + Global Attention Module	Image-level multi-class classification (Benign / Malignant / Normal)	100	96.76	96.08	-	91.78	91.84	NR

**Table 5:** Pooled diagnostic performance of imaging modalities, CNN architectures, learning types, and database sources

Dataset		Pooled SE (95% CI)	Pooled SP (95% CI)	SE_Var_StudyID	SE_Var_TableID	SP_Var_StudyID	SP_Var_TableID	AUC (95% CI)	Threshold_Spearman	Number of studies	Number of tables	Number of corrected tables
Whole_data		0.949 (0.922–0.967)	0.95 (0.909–0.973)	0.5483	0.5067	1.5275	0.3752	0.974 (0.961–0.981)	-0.382	20	45	6
Imaging modalities	Histopathology	0.979 (0.966–0.987)	0.934 (0.829–0.976)	0.0003	0.3782	0.1975	2.1925	0.981 (0.969–0.988)	-0.765	8	9	1
	Ultrasound	0.891 (0.846–0.923)	0.951 (0.836–0.986)	0.1223	0.0474	2.8801	0.0414	0.922 (0.856–0.96)	0.047	7	19	1
	CT	0.914 (0.803–0.965)	0.975 (0.863–0.996)	0.4861	0.6192	2.6316	0.3157	0.983 (0.957–0.987)	-0.051	4	11	1
	MRI	0.97 (0.89–0.992)	0.955 (0.909–0.978)	0	1.801	0	0.5629	0.986 (0.976–0.988)	0.309	1	6	3
CNN architectures	ResNet	0.92 (0.872–0.952)	0.947 (0.88–0.977)	0.0008	0.8056	1.6957	0	0.969 (0.921–0.98)	0.319	10	13	1
	DenseNet	0.927 (0.817–0.973)	0.932 (0.772–0.982)	0.9098	0	1.8355	0.047	0.956 (0.838–0.983)	-0.607	4	7	1
	Other*	0.967 (0.935–0.983)	0.945 (0.868–0.979)	0.8975	0.4237	2.0728	0.4494	0.979 (0.967–0.985)	-0.622	11	25	4
Learning type algorithms	Fully	0.959 (0.898–0.984)	0.929 (0.749–0.983)	1.2509	0.0708	2.5495	0.7631	0.962 (0.9–0.984)	-0.279	6	11	
	Transfer	0.942 (0.907–0.964)	0.948 (0.906–0.972)	0.3985	0.6729	1.1716	0.2335	0.978 (0.967–0.984)	-0.438	15	34	6
Type of databases	Open source	0.98 (0.957–0.991)	0.971 (0.853–0.995)	0.3333	0.3333	2.1925	2.1925	0.985 (0.963–0.989)	-0.765	6	6	1
	Non_open source	0.931 (0.895–0.956)	0.938 (0.89–0.967)	0.3956	0.4687	1.0192	0.37	0.968 (0.954–0.978)	-0.299	14	39	5

* Other CNN architectures included: DCNN, GoogLeNet, MobileNet, VGG16, Xception, Inception, hybrid models like Inception-ResNet v2, and CNN & GoogleNet V3

AUC: Area Under the Curve, CI: Confidence Interval, CNN: Convolutional Neural Network, CT: Computed Tomography, MRI: Magnetic Resonance Imaging, SE: Sensitivity, SP: Specificity, Var: Variance

### Pooled performance of CNNs algorithms

**Fig. 2:**
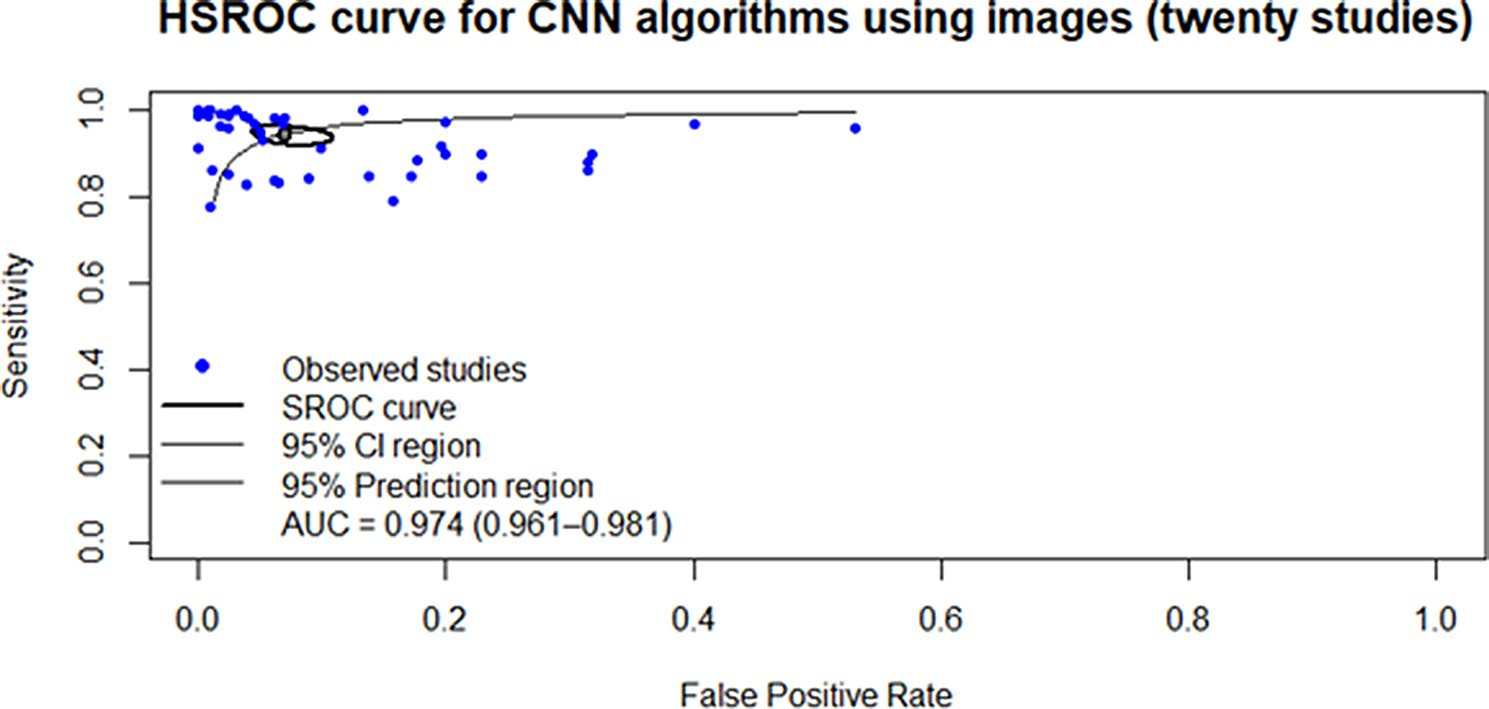
Hierarchical summary receiver operating characteristic of all studies included in the meta-analysis

A multilevel random-effects model was used to pool SE (0.94, 95% CI: 0.92–0.96) and SP (0.95, 95% CI: 0.90–0.97) separately. Additionally, a HSROC model was applied to estimate the summary ROC curve, yielding an AUC of 0.974 (95% CI: 0.961–0.981),(Table 5; Fig. 2).

**Fig. 3:**
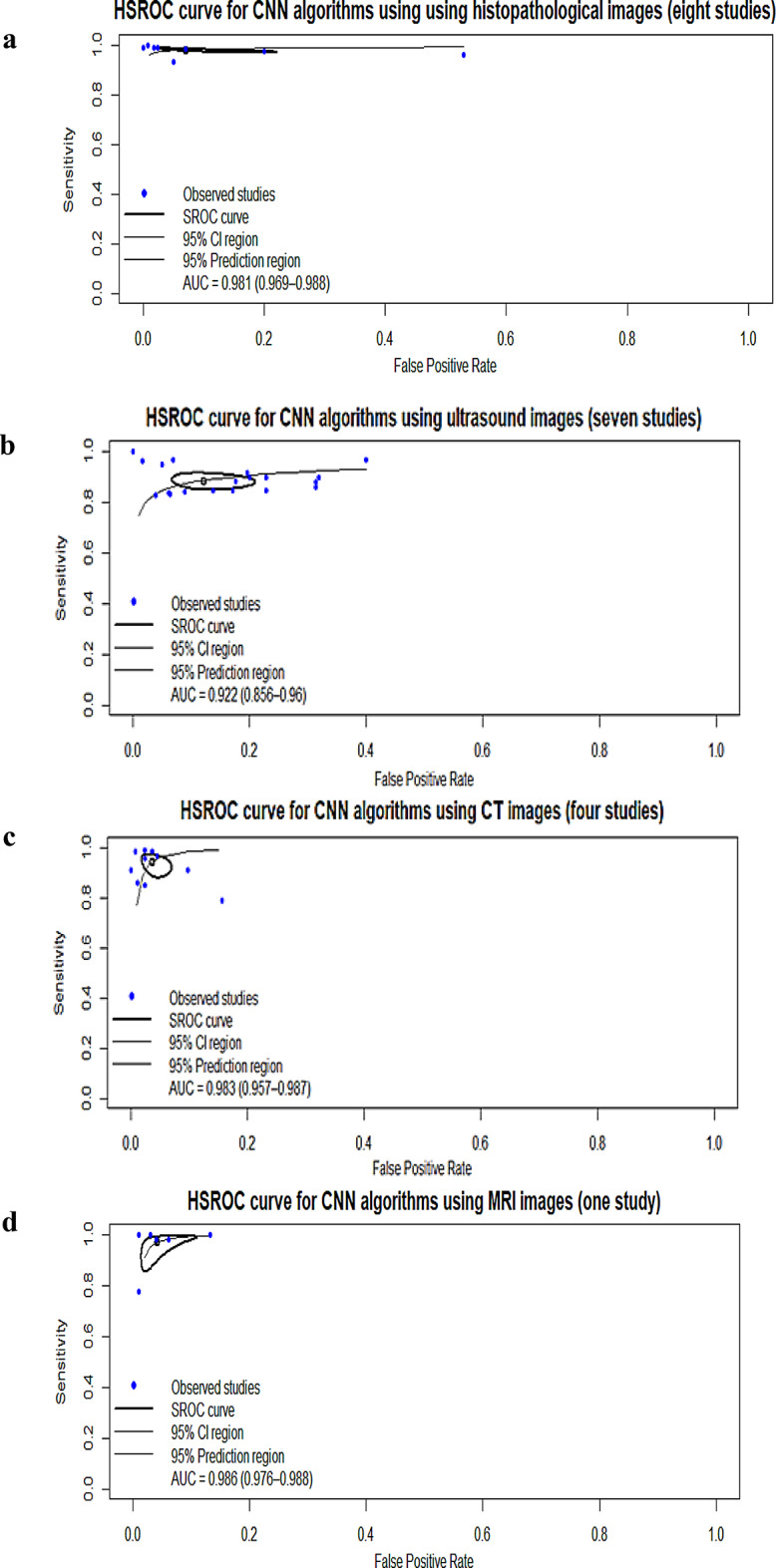
(**a**) Hierarchical summary receiver operating characteristic of studies using histopathological images. (**b**) Hierarchical summary receiver operating characteristic of using ultrasound images. (**c**) Hierarchical summary receiver operating characteristic of studies using computed tomography images. (**d**) Hierarchical summary receiver operating characteristic of studies using magnetic resonance imaging

Subgroup analyses revealed variability by imaging modality, CNN architecture, learning type, and database type. Histopathology studies (*n* = 8) showed the highest accuracy (SE, 0.979; SP, 0.934; AUC, 0.981) [18, 19, 34, 36, 52, 53, 55, 57] (Table 5; Fig. 3a and Figure S1a–b), followed by ultrasound studies (7 studies; SE, 0.891; SP, 0.951; AUC, 0.922) [7, 25, 59, 61–63, 69](Table 5; Fig. 3b and Figure S1a–b). CT studies (4 studies; SE, 0.914; SP, 0.975; AUC, 0.983) [71, 76, 78, 80] (Table 5; Fig. 3c and Figure S1a–b), and the MRI study (1 study; SE, 0.97; SP, 0.955; AUC, 0.986) [85] (Table 5; Fig. 3d and Figure S1a–b).

**Fig. 4:**
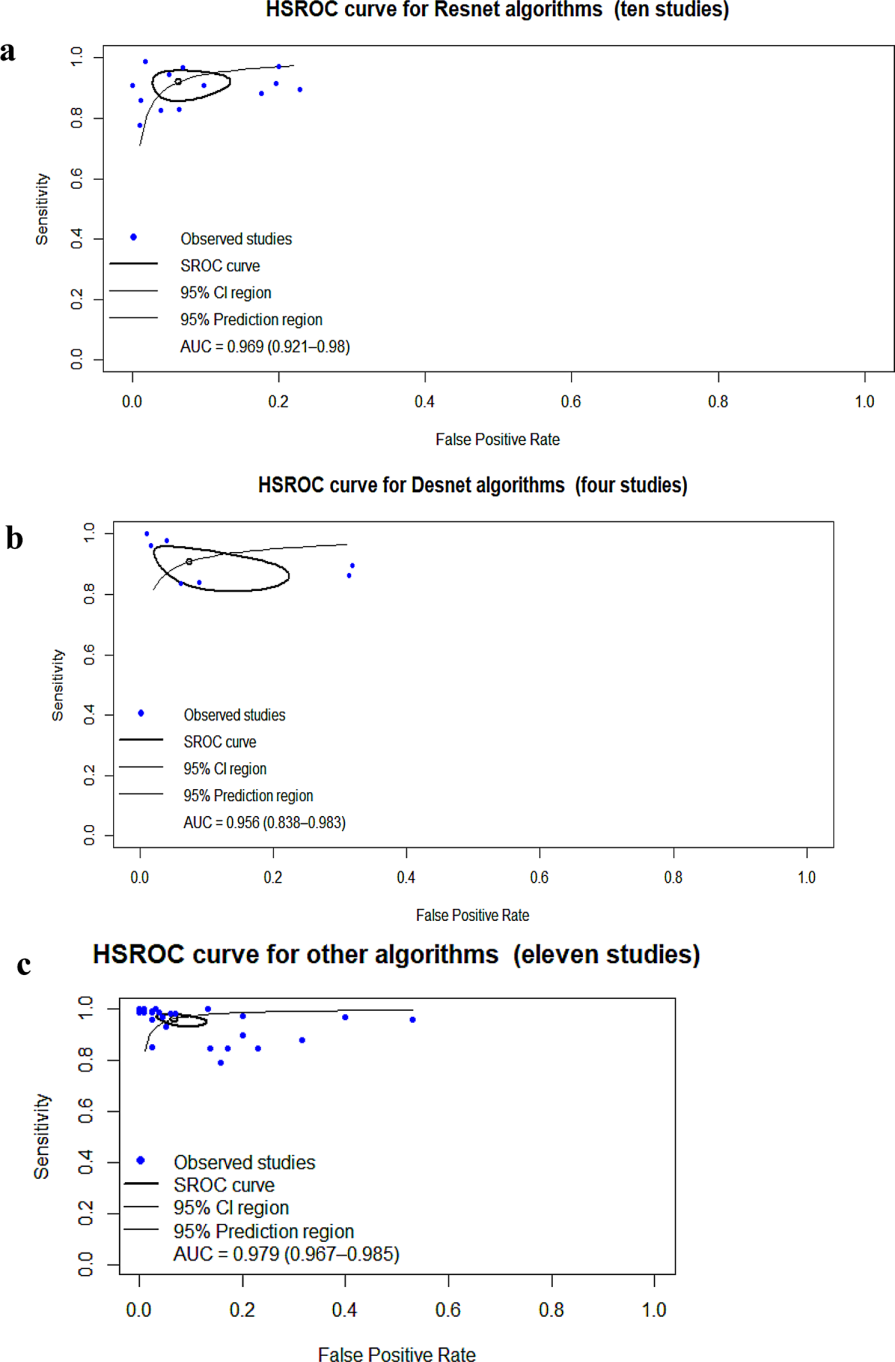
(**a**) Hierarchical summary receiver operating characteristic of studies using ResNet algorithms. (**b**) Hierarchical summary receiver operating characteristic of studies using Densenet algorithms. (**c**) Hierarchical summary receiver operating characteristic of studies using other algorithms (AlexNet, DCNN, DFCNN, GoogLeNet, Hybrid (CNN & GoogLeNet V3), Hybrid (Inception-ResNet v2), Hybrid CNN–Transformer model, InceptionV3, NASNetLarge, NASNetMobile, OVANet (Ensemble CNN: VGG19 + InceptionV3 + SE + spatial attention), OvCan-FIND, MobileNet, VGG16, VGG19, Xception, Xception_ViT)

For CNN architectures, ResNet studies (10 studies) achieved SE, 0.92; SP, 0.947; AUC, 0.969 [25, 34, 36, 59, 61, 62, 76, 78, 80, 85] (Table 5; Fig. 4a and Figure S2a–b). DenseNet studies (4 studies) achieved SE, 0.927; SP, 0.932; AUC, 0.956 [7, 59, 63, 85] (Table 5; Fig. 4b and Figure S2a–b), and other architectures—including AlexNet, DCNN, DFCNN, GoogLeNet, Hybrid (CNN & GoogLeNet V3), Hybrid (Inception-ResNet v2), Hybrid CNN–Transformer model, InceptionV3, NASNetLarge, NASNetMobile, OVANet (Ensemble CNN: VGG19 + InceptionV3 + SE + spatial attention), OvCan-FIND, MobileNet, VGG16, VGG19, Xception, Xception_ViT (11 studies)—achieved SE, 0.967; SP, 0.945; AUC, 0.979 [18, 19, 52, 53, 55, 57, 59, 69, 71, 80, 85] (Table 5; Fig. 4c and Figure S2a–b).

**Fig. 5:**
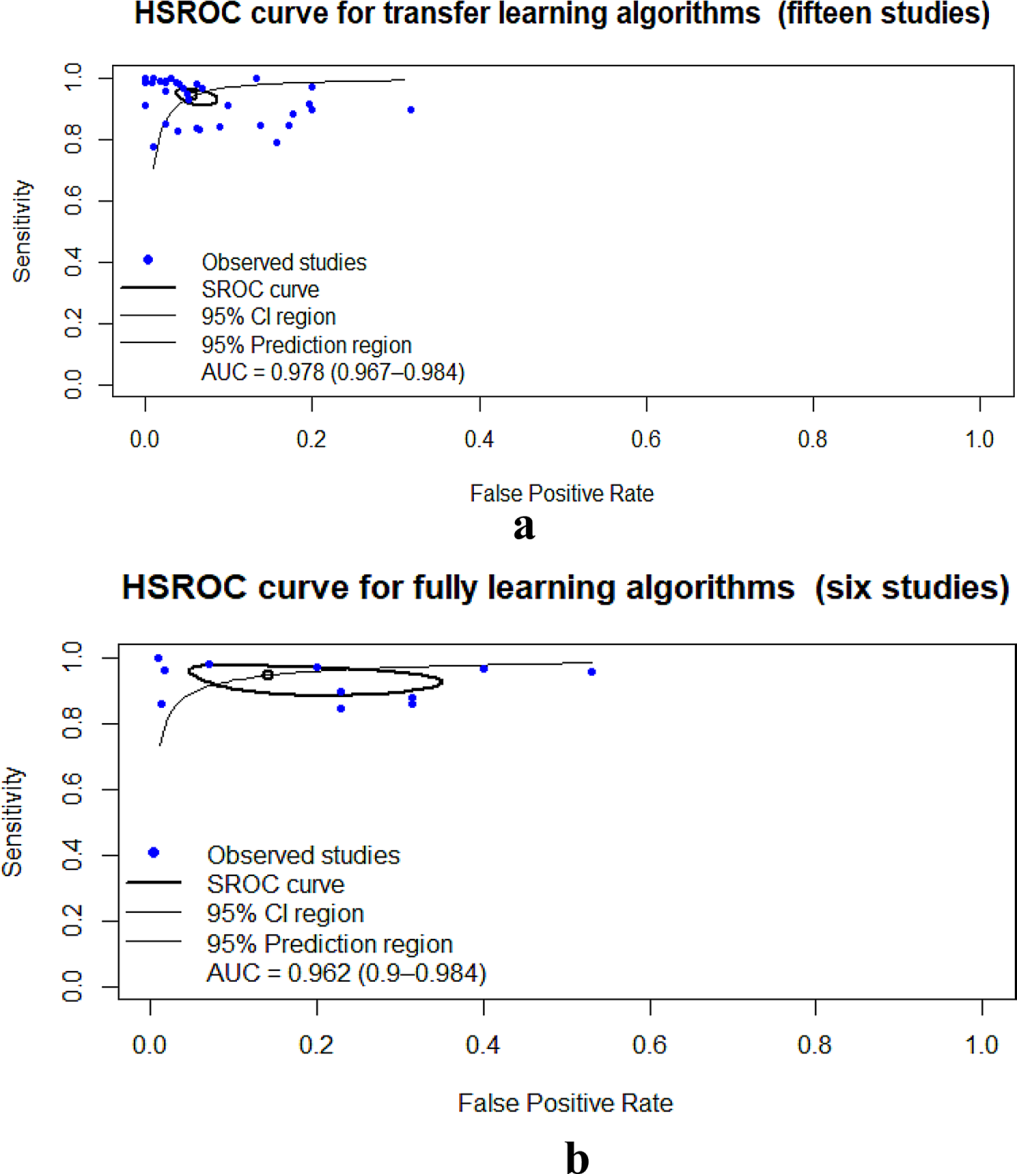
(**a**) Hierarchical summary receiver operating characteristic of studies used transfer learning algorithms. (**b**) Hierarchical summary receiver operating characteristic of studies using fully learning algorithms

Transfer learning studies (15 studies) had SE, 0.942; SP, 0.948; AUC, 0.978 [7, 25, 34, 36, 52, 55, 57, 59, 61, 62, 69, 71, 78, 80, 85] (Table 5; Fig. 5a and Figure S3a–b), fully trained studies (6 studies) had SE, 0.959; SP, 0.929; AUC, 0.962 [18, 19, 53, 59, 63, 76] (Table 5; Fig. 5b and Figure S3a–b).

**Fig. 6:**
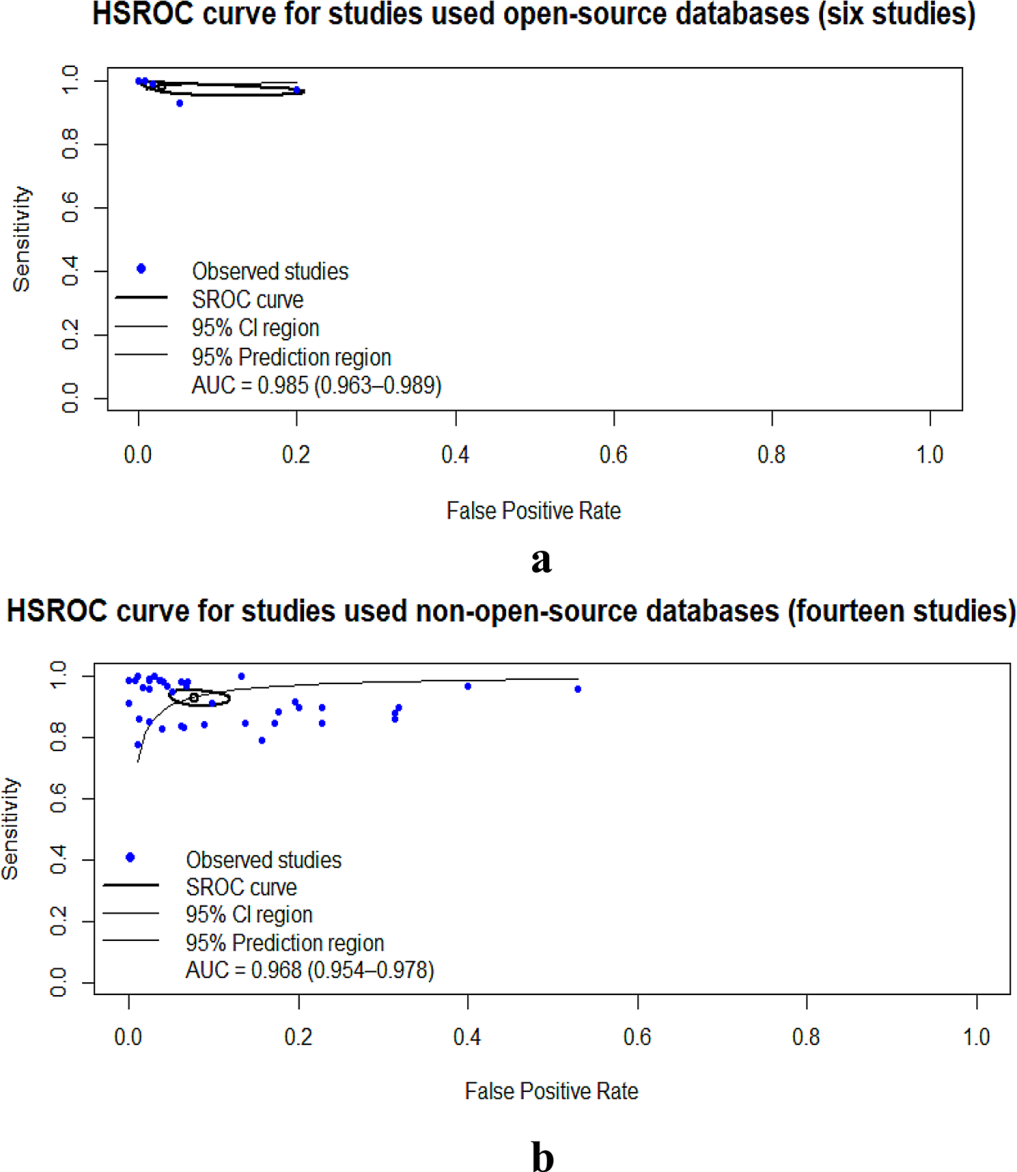
(**a**) Hierarchical summary receiver operating characteristic of studies used open-source datasets. (**b**) Hierarchical summary receiver operating characteristic of studies used non- open-source datasets

Open-source dataset studies (6 studies) yielded SE, 0.98; SP, 0.971; AUC, 0.985 [18, 19, 34, 36, 52, 69](Table 5; Fig. 6a and Figure S4a–b), whereas non-open-source dataset studies (14 studies) showed SE, 0.931; SP, 0.938; AUC, 0.968 [7, 25, 53, 55, 57, 59, 61–63, 71, 76, 78, 80, 85] (Table 5; Fig. 6b and Figure S4a–b).

### Heterogeneity analysis

Heterogeneity across studies was assessed using variance in SE and SP (Table 5). Overall, SE variance was 0.548 and SP variance 1.527, indicating substantial variability in model performance. Among imaging modalities, CT showed the highest heterogeneity, while MRI and ultrasound were more consistent. Regarding CNN architectures, the “Other” group exhibited the greatest variability, whereas DenseNet was the most stable. Fully supervised learning models were relatively homogeneous, while Transfer learning and unreported algorithms showed higher heterogeneity. Open-source datasets demonstrated lower variance compared to non-open-source datasets. These results indicate that heterogeneity is influenced by imaging modality, CNN architecture, learning algorithm, and data source, and should be considered when interpreting the findings.

### Subgroup analysis

**Fig. 7:**
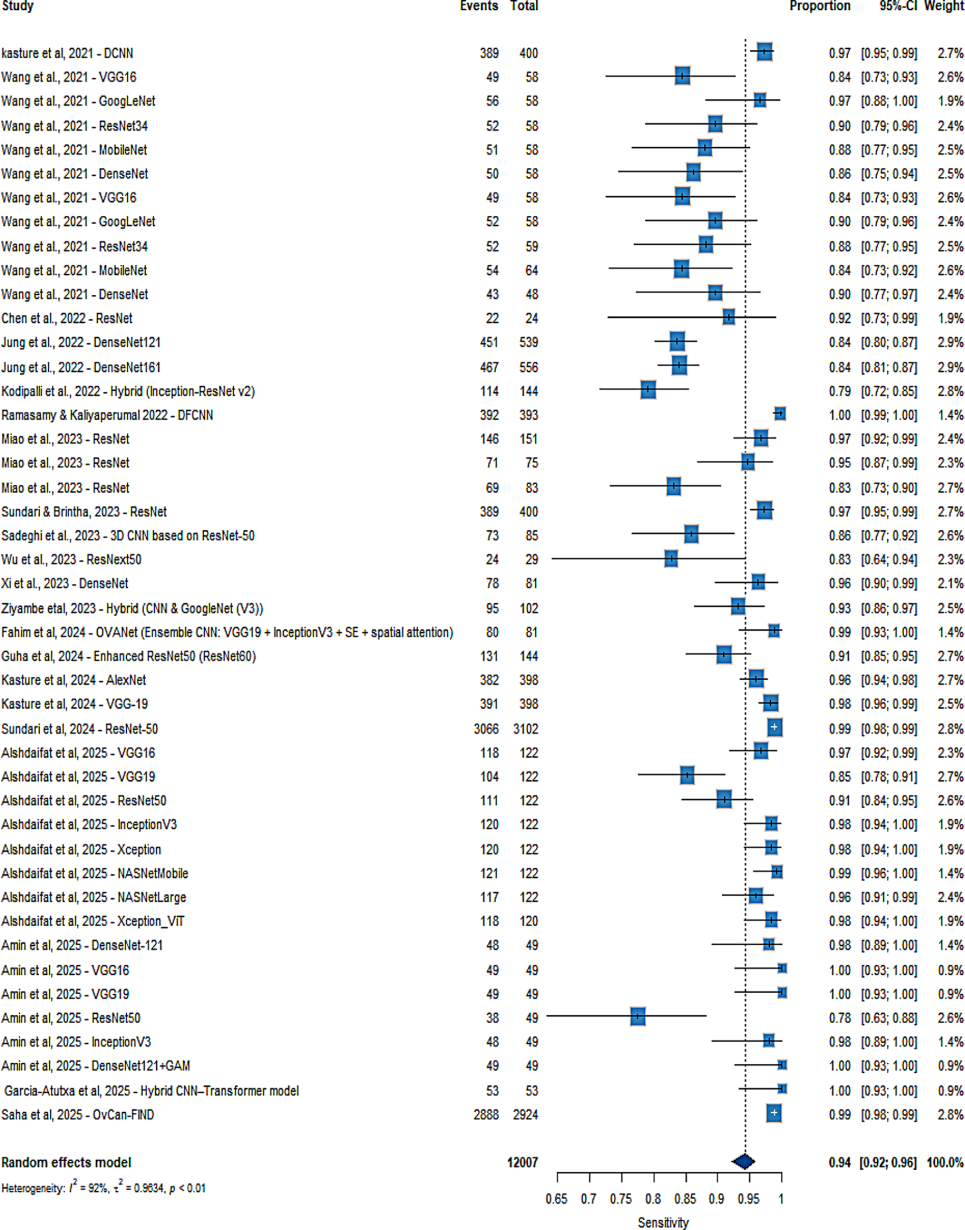

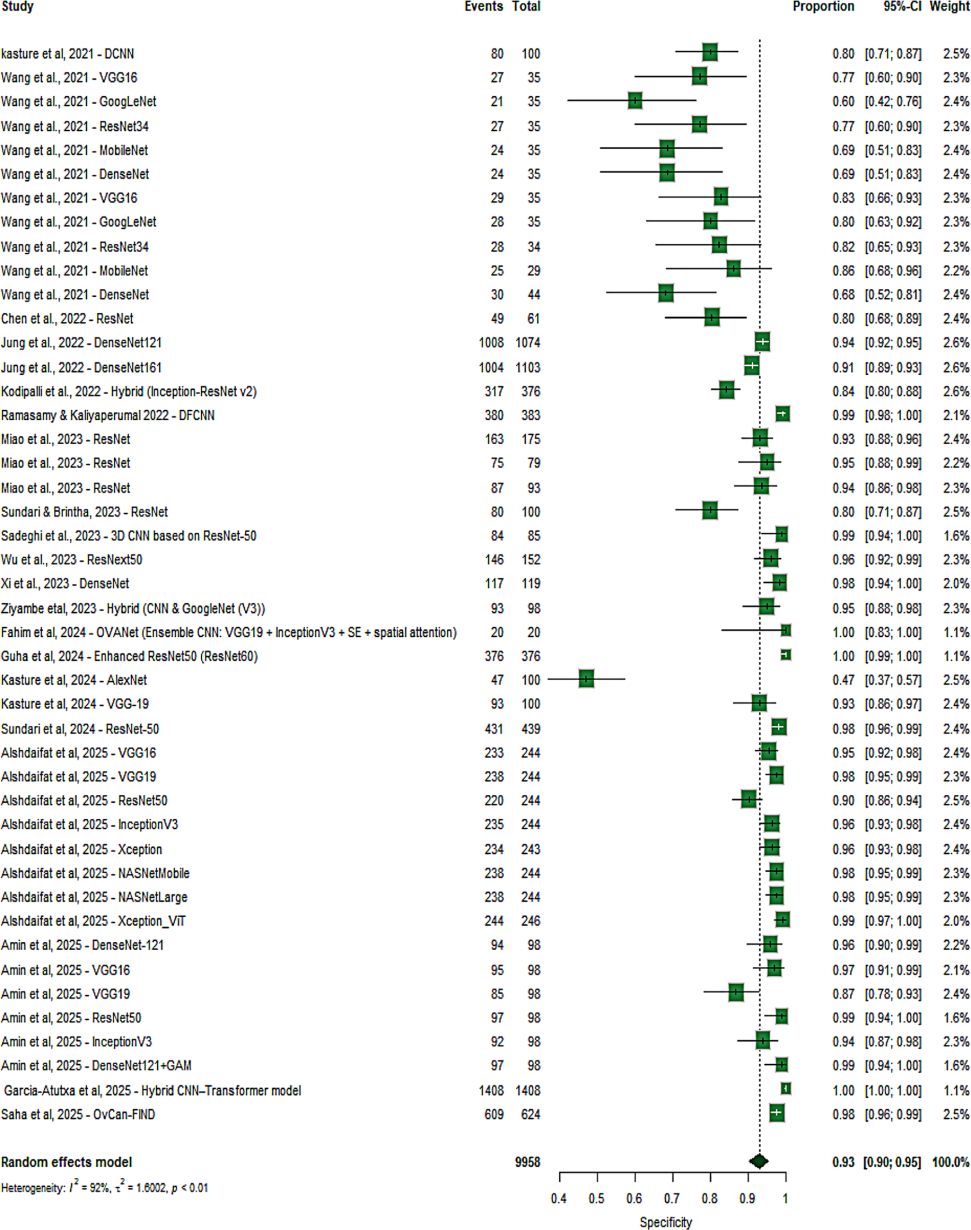
Forest plot of studies included in the meta-analysis (20 studies, 45 tables). There was substantial heterogeneity, with sensitivity and specificity an I² of 92% (*p* < 0.01)

To further explore heterogeneity, I² statistics were calculated across all subgroups. Regarding imaging modalities, high heterogeneity in SE was observed for histopathology (I² = 82%), CT (I² = 83%), and MRI (I² = 77%), while ultrasound demonstrated moderate heterogeneity (I² = 54%). For SP, heterogeneity was high across all modalities, particularly for histopathology (I² = 96%), followed by CT (I² = 90%), ultrasound (I² = 88%), and MRI (I² = 71%). Concerning CNN architectures, ResNet showed substantial heterogeneity in SE (I² = 93%), whereas DenseNet demonstrated comparatively lower heterogeneity (I² = 65%); other models also showed high variability (I² = 90%). For SP, heterogeneity remained high across architectures, including ResNet (I² = 85%), DenseNet (I² = 91%), and other models (I² = 93%). In terms of training strategy, transfer learning studies exhibited higher heterogeneity in SE (I² = 93%) compared to fully trained models (I² = 84%), while SP heterogeneity was greater in fully trained models (I² = 92%) than in transfer learning approaches (I² = 87%). Across database types, open-source datasets showed lower heterogeneity for SE (I² = 82%) compared to non-open datasets (I² = 90%), but higher heterogeneity for SP (I² = 94% vs. 91%) (Figure S1–S4). Overall, heterogeneity remained high across all subgroups, indicating that factors such as imaging modality, CNN algorithm, learning type, and database type may have influenced performance (Fig. 7).

### Meta-regression analysis (*n* = 20, k = 45)

Multivariate random-effects meta-regression (REML) was conducted on 45 observations. The overall test of moderators was significant (Wald χ²(7) = 14.96, *p* = 0.0365). Among the included predictors, only the “Other” algorithm family showed a significant association with logDOR, demonstrating higher performance compared with ResNet (coef = 0.987, *p* = 0.0011). Imaging modality (CT, MRI, Ultrasound), dataset openness, transfer learning, and DenseNet were not significantly associated with logDOR (all *p* > 0.05). The intercept, representing Histopathology + ResNet + fully supervised learning + non-open datasets, was 4.241 (*p* = 0.0013). Residual heterogeneity remained high (τ² = 4.993; I² ≈ 91%), and the model explained approximately 12.8% of the between-study variance (Supplementary Table 1).

### Meta-regression analysis (large studies *n* = 7, k = 9)

In large-sample studies (*n* ≥ 300; k = 9), multivariate random-effects meta-regression did not identify any statistically significant moderators of log diagnostic odds ratio (logDOR) (QM(df = 5) = 4.89, *p* = 0.429). Imaging modality, dataset type (open vs. non-open), learning strategy, CNN architecture, and task type were not significantly associated with logDOR (all *p* > 0.05). However, residual heterogeneity remained substantial (τ² = 5.86), and the test for residual heterogeneity was significant (QE(df = 3) = 74.18, *p* < 0.001), indicating considerable unexplained between-study variability even among large studies (Supplementary Table 2).

### Leave-one-out se analysis

The pooled logDOR was stable across individual study removals. The largest changes in the pooled estimate were observed for the studies with the highest and lowest individual effect sizes [57, 69] (see Supplementary Table 3). Standard errors remained consistent across all removals, confirming the robustness and reliability of the meta-analysis results.

### Quality assessment

The quality of the studies included in the review was assessed using QUADAS-2 with the results summarized in Figure S5a (bar plot) and Figure S5b (traffic lights). A thorough evaluation of each domain focusing on risk of bias was conducted. For patient selection, 28 studies were classified as having high risk of bias or some concerns, mainly due to reporting limitations or inappropriate application of inclusion and exclusion criteria. In the index test domain, 23 studies were rated as having high risk or some concerns, while the remaining studies were considered low risk. In the reference standard domain, 22 studies demonstrated high risk of bias or some concerns. For flow and timing, 27 studies were judged to have high risk or some concerns, primarily due to insufficient reporting of time intervals between the index test and reference standard or lack of clarity regarding whether all participants received the same reference standard.

Deeks’ test revealed a significant asymmetry in the funnel plot (Slope = -39.268, *p* = 0.0115), suggesting the presence of potential publication bias in the included studies. The negative slope indicates that smaller studies tended to report higher logDOR values compared to larger studies, which is consistent with selective reporting or small-study effects. Therefore, the meta-analytic estimate of diagnostic performance should be interpreted with caution, as it may be influenced by this bias (Figure S6).

## Discussion

CNNs, as state-of-the-art image analysis tools, can learn complex features from images to aid in diagnosis, prognosis, and subtyping. Multi-modal approaches, combining different imaging types and clinical data, improve generalizability and performance across cancer types [27, 86, 87]. This systematic review aimed to evaluate the diagnostic performance of CNN algorithms for ovarian cancer detection. A total of 47 studies were included, of which 20 studies contributed data for meta-analysis. CNNs demonstrated strong pooled diagnostic performance with a SE of 94% and SP of 95%, and an AUC of 0.974. Performance varied by imaging modality: histopathology achieved high diagnostic accuracy (SE 97.9%, SP 93.4%, AUC 0.981), followed by MRI (SE 97%, SP 95.5%, AUC 0.986), CT (SE 91.4%, SP 97.5%, AUC 0.983), and ultrasound (SE 89.1%, SP 95.1%, AUC 0.922). However, the MRI estimate was based on a single study. These findings underscore the importance of selecting appropriate imaging techniques for CNN-based ovarian cancer detection.

The performance of CNNs varied across studies, as exemplified by Urushibara, et al. (2022), who reported high diagnostic performance in diagnosing endometrial cancer on MRI. Specifically, a deep learning model using CNNs outperformed expert radiologists when analyzing axial apparent diffusion coefficient maps and axial contrast-enhanced T1-weighted images. Although the addition of other image types to the training data improved diagnostic performance for some image sets, this improvement was not statistically significant [88]. The variability in CNN performance may be related to the quality of the images used. The exclusion of low-quality images in some studies and the absence of image quality reporting in others suggest variability in data quality, which may have contributed to the observed heterogeneity. The transferability of CNN models to data from different sources and the identification of uncertain predictions remain significant challenges. The role of tissue quality itself is also largely unknown. In this study, we demonstrated that samples from The Cancer Genome Atlas ovarian cancer dataset (TCGA-OV) with different tissue sources exhibit varying quality characteristics, and CNN performance is linked to this property. In Mayer et al., study CNNs performed best on high-quality data. While quality control tools were partially effective in identifying low-quality tiles, their use did not enhance the performance of the trained CNNs. To address this, they trained NoisyEnsembles by introducing label noise during training, which improved CNN performance on low-quality, unknown datasets. The performance of the NoisyEnsembles increased as the ensemble became more consistent, suggesting that incorrect predictions could be efficiently discarded to avoid erroneous diagnostic decisions [89].

Among the CNN architectures evaluated, ResNet and DenseNet demonstrated strong diagnostic performance, with pooled AUCs of 0.969 and 0.956, respectively, while other CNN architectures and hybrid models showed slightly higher overall performance. Transfer-learning approaches outperformed fully trained models, showing higher pooled SE and SP, underlining the value of transfer learning in improving CNN performance. Transfer learning is particularly beneficial in ovarian cancer detection when datasets are limited or heterogeneous: by fine-tuning pre-trained models, the network can leverage strong low- and mid-level features, bolstering its discriminative ability without requiring very large medical datasets [90]. For instance, Wu, et al. used six pre-trained DCNNs (including ResNext50, ResNet34, DenseNet121) on ultrasound images of ovarian tumors and achieved an accuracy of 0.952, with 90% SE and 99.2% SP for high-grade serous carcinoma [62]. Similarly, an ensemble of transfer‑learned CNNs (VGG16, ResNet50, and InceptionNet) attained a SE of 96.5% and SP of 88.1% in classifying benign vs. malignant ovarian masses [91].

Substantial heterogeneity was observed across the included studies, with SE and SP variances of 0.548 and 1.528, respectively, indicating notable variability in CNN diagnostic performance across different study settings and methodological characteristics. Multivariate random-effects meta-regression indicated that the overall set of moderators was significant. Among the evaluated predictors, only the “Other” CNN architecture group was significantly associated with logDOR, demonstrating higher diagnostic performance compared with ResNet. In contrast, imaging modality (CT, MRI, and ultrasound), dataset openness, transfer learning, and DenseNet architecture were not significantly associated with logDOR. Despite these analyses, the included covariates explained only about 12.8% of the between-study variance, and substantial residual heterogeneity remained, suggesting that additional unmeasured factors may contribute to variability in CNN diagnostic performance.

In the present study, the quality of the included studies was assessed using the QUADAS-2 tool. However, during the review, two additional checklists designed for AI in medical imaging were identified: CLAIM (Checklist for Artificial Intelligence in Medical Imaging) and STARD-AI (Standards for Reporting of Diagnostic Accuracy Studies – Artificial Intelligence). CLAIM is a descriptive checklist comprising 17 domains and 44 items, providing comprehensive guidelines for reporting AI studies in medical imaging. However, it lacks a quantitative scoring system to assess the risk of bias. To improve its utility, CLAIM needs to be updated to include a scoring system [92]. STARD-AI, still in preparation, aims to standardize the reporting of diagnostic accuracy studies involving AI-based tests [93]. To make these checklists more effective, it is essential to introduce a scoring system that quantifies the degree to which each item is addressed. This approach allows for an objective assessment of study quality and potential biases, ultimately leading to more consistent and reliable evaluations.

### Limitations and future directions

This systematic review and meta-analysis has several limitations. First, the included studies showed considerable heterogeneity in imaging modalities, CNN architectures, preprocessing methods, and reporting standards, which may affect comparability and pooled estimates. Second, only 20 out of 47 studies provided sufficient data for inclusion in the meta-analysis, potentially limiting statistical power. Third, most studies were single-center or region-specific, which may limit the generalizability of CNN models across diverse patient populations and geographic regions. Variations in imaging protocols, equipment, and population characteristics could influence model performance in broader clinical settings. Finally, although meta-regression was performed, the included covariates explained only a small proportion of the between-study variance, suggesting that additional unmeasured factors may contribute to the observed heterogeneity. Regarding performance evaluation, most studies reported standard metrics such as SE, SP, and AUC. While these are informative, additional metrics like Matthews Correlation Coefficient (MCC) or Precision-Recall AUC could provide deeper insights, particularly in clinical contexts where false negatives are critical. Incorporating such metrics in future studies would improve the assessment of model reliability and clinical applicability.

Future work should focus on standardizing data collection, image quality control, and algorithm selection protocols to minimize variability. Validation across multi-center and multi-national datasets, as well as the use of multi-modal imaging and transfer learning techniques, could enhance the robustness, versatility, and generalizability of CNN-based approaches. Expanding applications to tasks such as metastasis detection or early-stage cancer screening may further broaden the clinical utility of these models. Finally, adherence to reporting frameworks like CLAIM and STARD-AI is recommended to improve study quality, reduce heterogeneity, and facilitate reproducibility.

## Conclusion

CNN-based algorithms demonstrate high diagnostic accuracy for ovarian cancer detection, with particularly strong performance across imaging modalities such as MRI, CT, and histopathology. These findings highlight the potential of deep learning models to support AI-assisted diagnostic workflows and improve early detection. However, substantial heterogeneity across studies and potential publication bias indicate the need for standardized imaging protocols, larger multi-center datasets, and external validation. Future research should focus on harmonizing data sources and integrating CNN-based tools into clinical decision-making to enhance diagnostic reliability and patient outcomes.

## Supplementary Information

Below is the link to the electronic supplementary material.

Supplementary Material 1

Supplementary Material 2

## Data Availability

The data supporting the findings of this study are available from the corresponding author upon reasonable request.
