# Supplementary material for: Diagnostic accuracy of ovarian cancer using convolutional neural network: a systematic review and meta-analysis
Source: BMC Med Inform Decis Mak. 2026 Apr 18;26:191. doi: 10.1186/s12911-026-03462-9 (PMC13217775; doi:10.1186/s12911-026-03462-9)
Supplement: Supplementary file 1 — Supplementary Material 1 [file 12911_2026_3462_MOESM1_ESM.docx]

Supplmentray Materials:

# Diagnostic Accuracy of Ovarian Cancer using Convolutional Neural Network: A Systematic Review and Meta-Analysis.

**Supplementary Figures 1-4:** Forest plot of different medical image modalities, applied algorithms, types of learning and source of databases

| 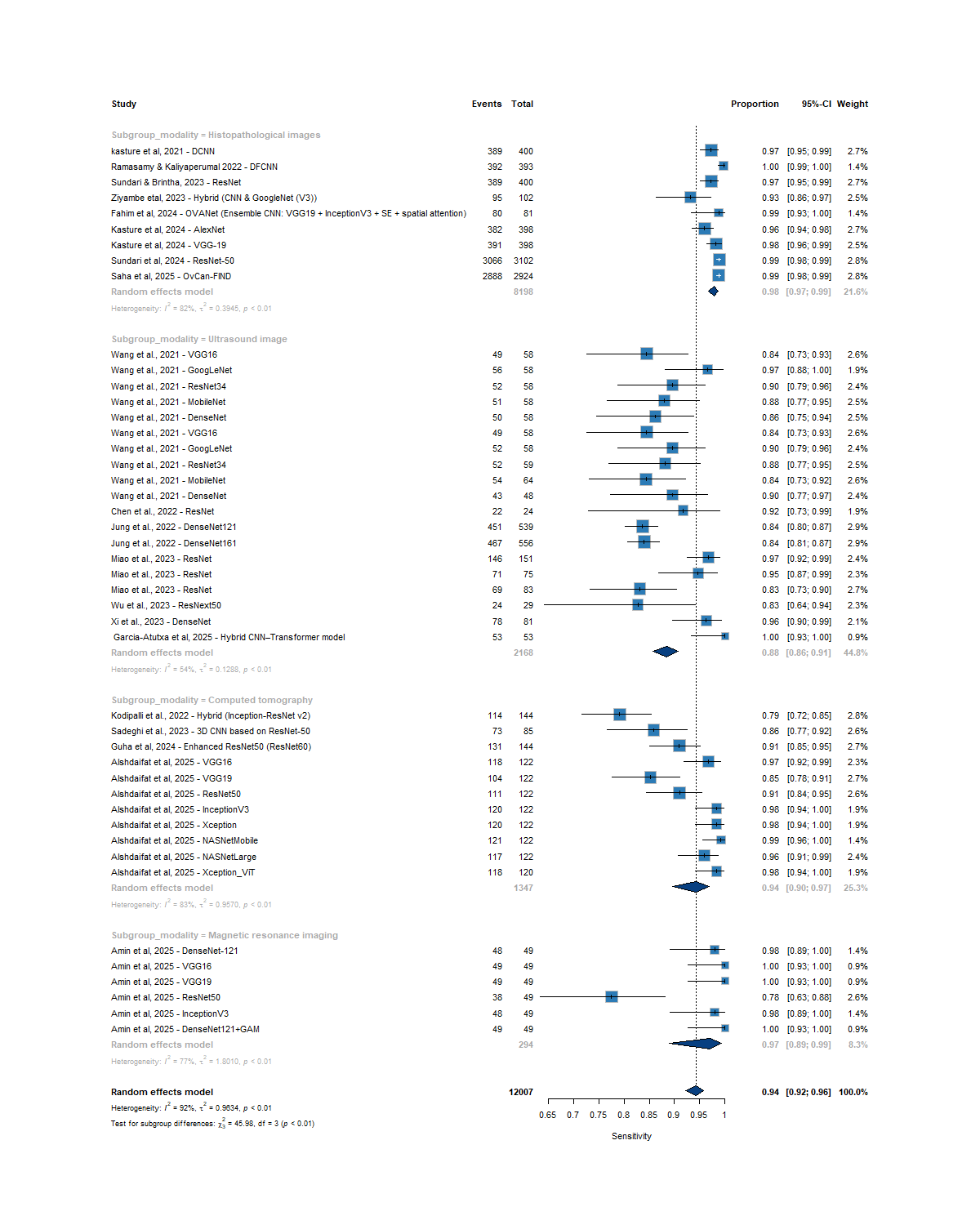 | 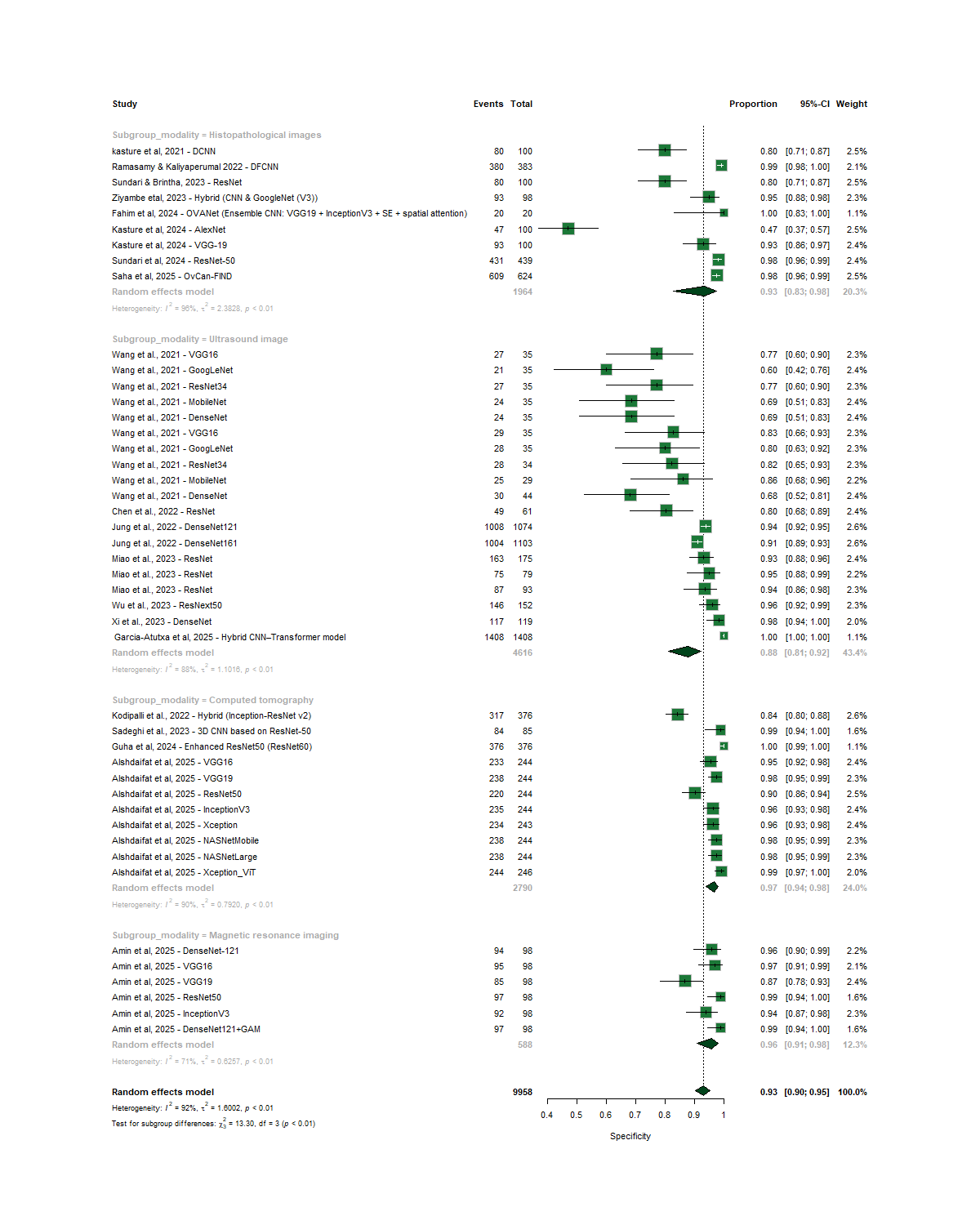 |
| --- | --- |
| **Figure S1a:** Sensitvity of studies based on the modalies in the meta-analysis (20 studies, 45 tables). | **Figure S1b:** Specificity of studies based on the modalies in the meta-analysis (20 studies, 45 tables). |
| 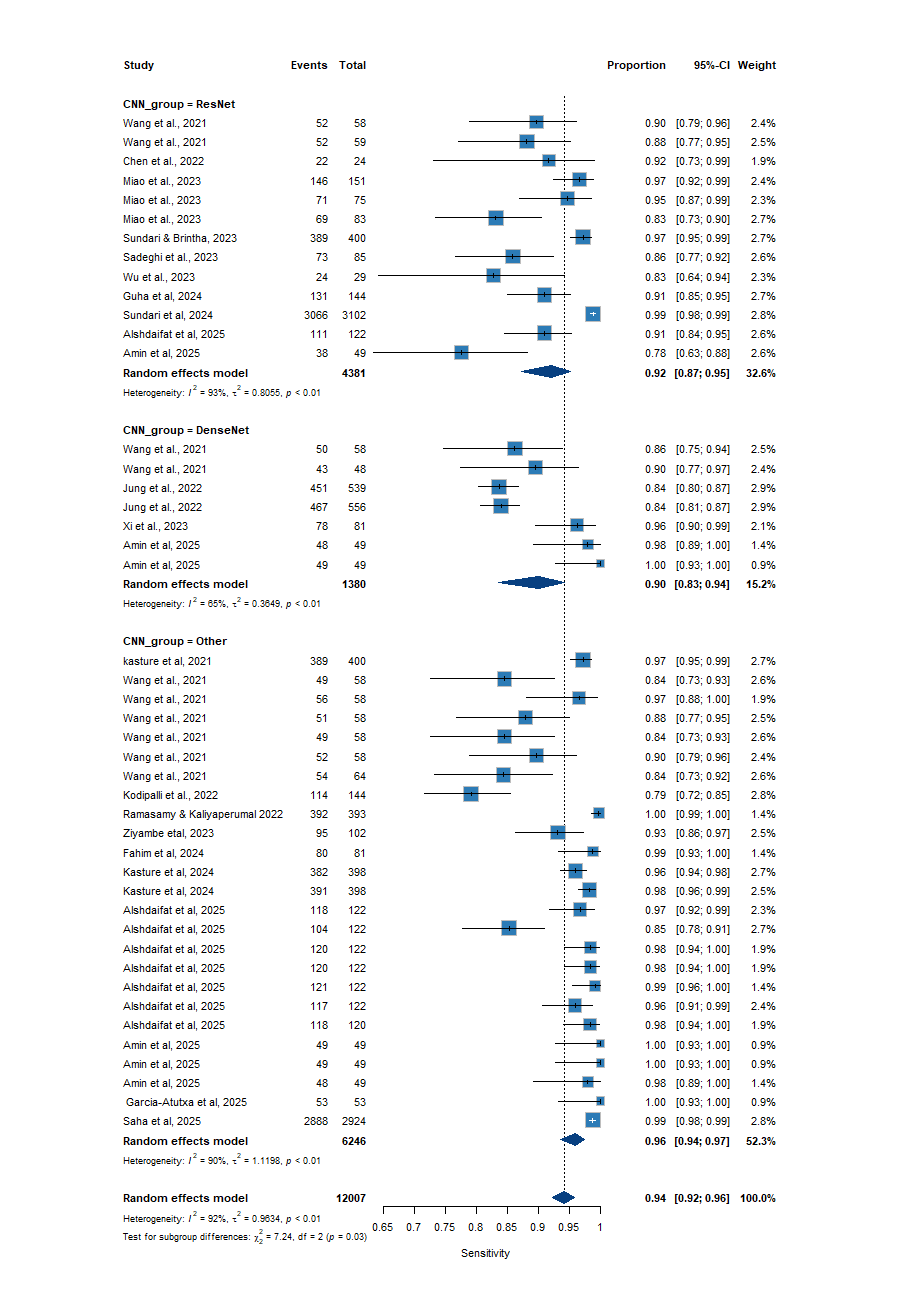 | 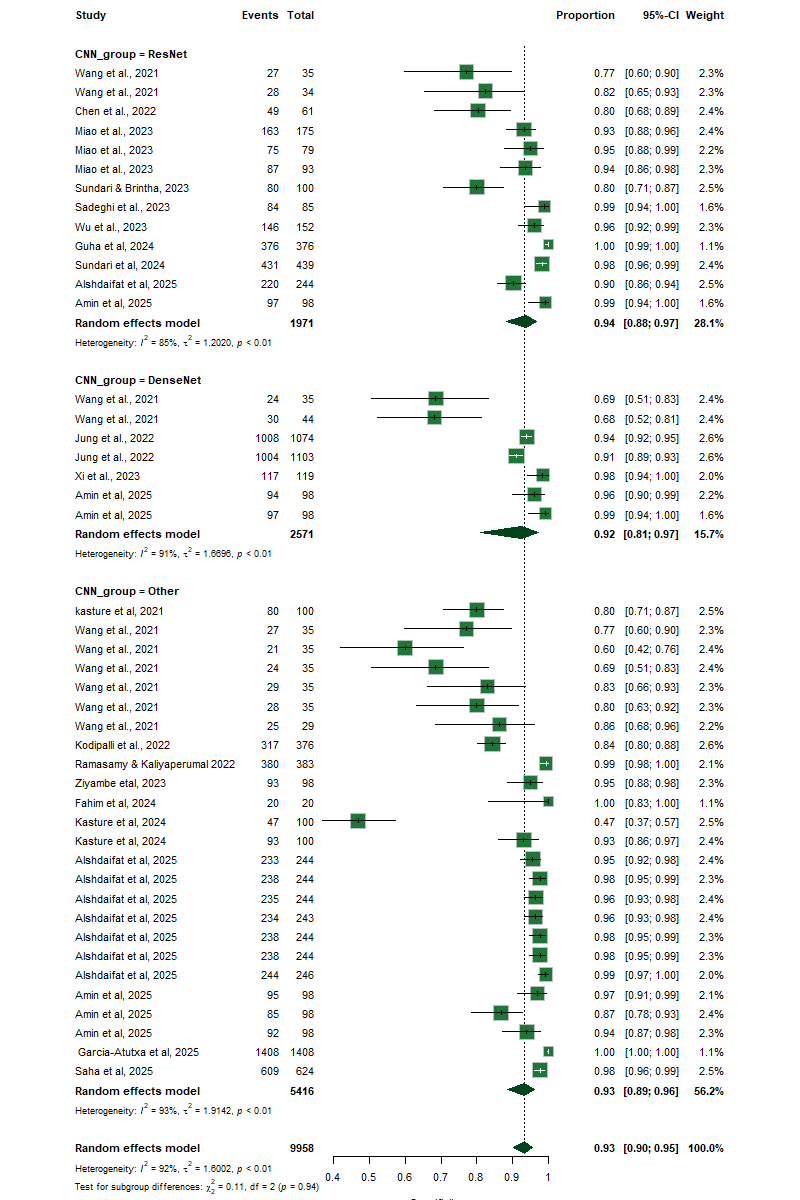 |
| **Figure S2a:** Sensitvity of studies based on the CNN algorithms in the meta-analysis (20 studies, 45 tables). | **Figure S2b:** Specificity of studies based on the CNN algorithms in the meta-analysis (20 studies, 45 tables)). |
| 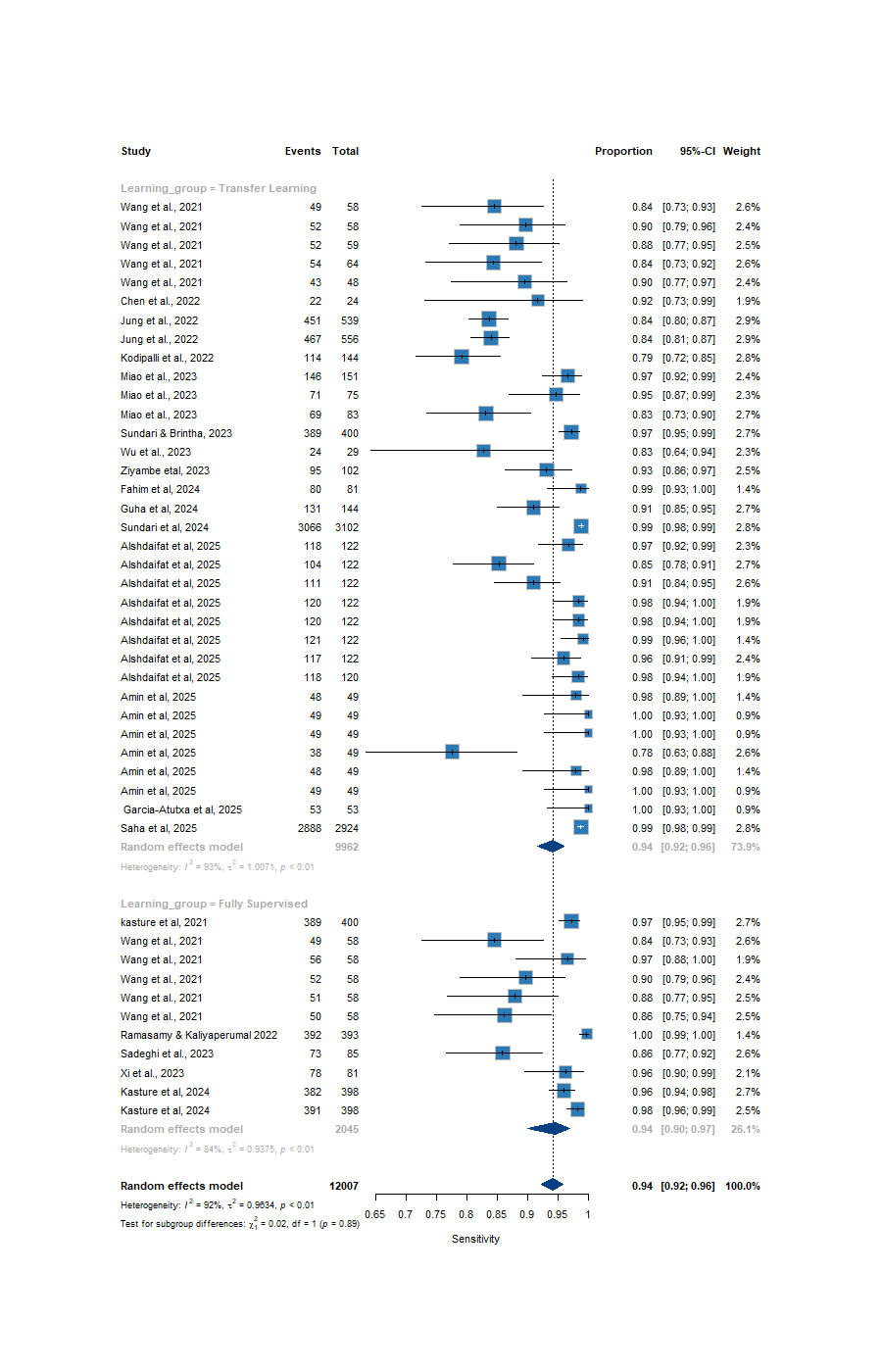 | 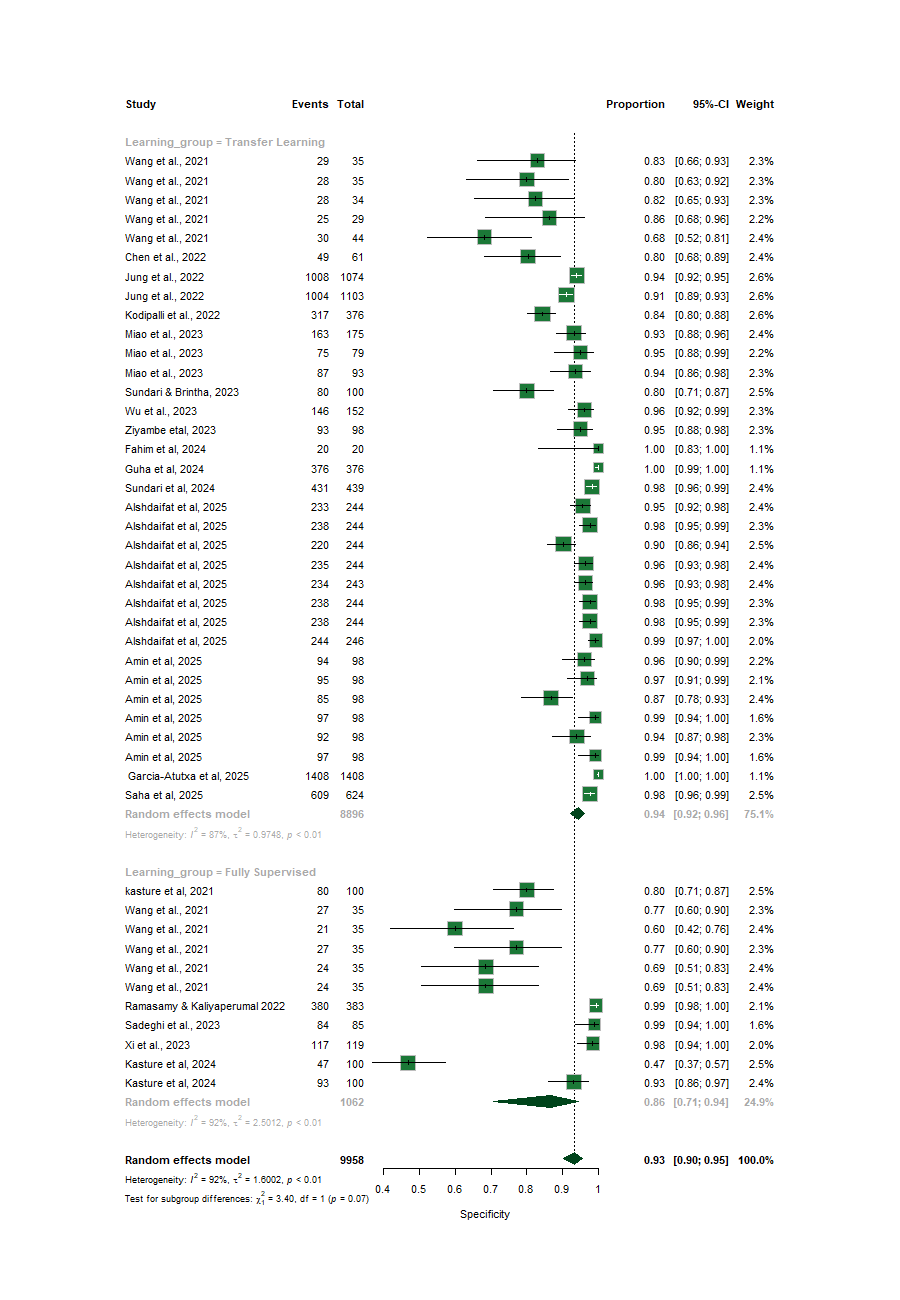 |
| **Figure S3a:** Sensitvity of studies applied transfer/fully/not reported learning algorithms (20 studies, 45 tables). | **Figure S3b:** Specificity of studies applied transfer/fully/not reported learning algorithms (20 studies, 45 tables). |
| 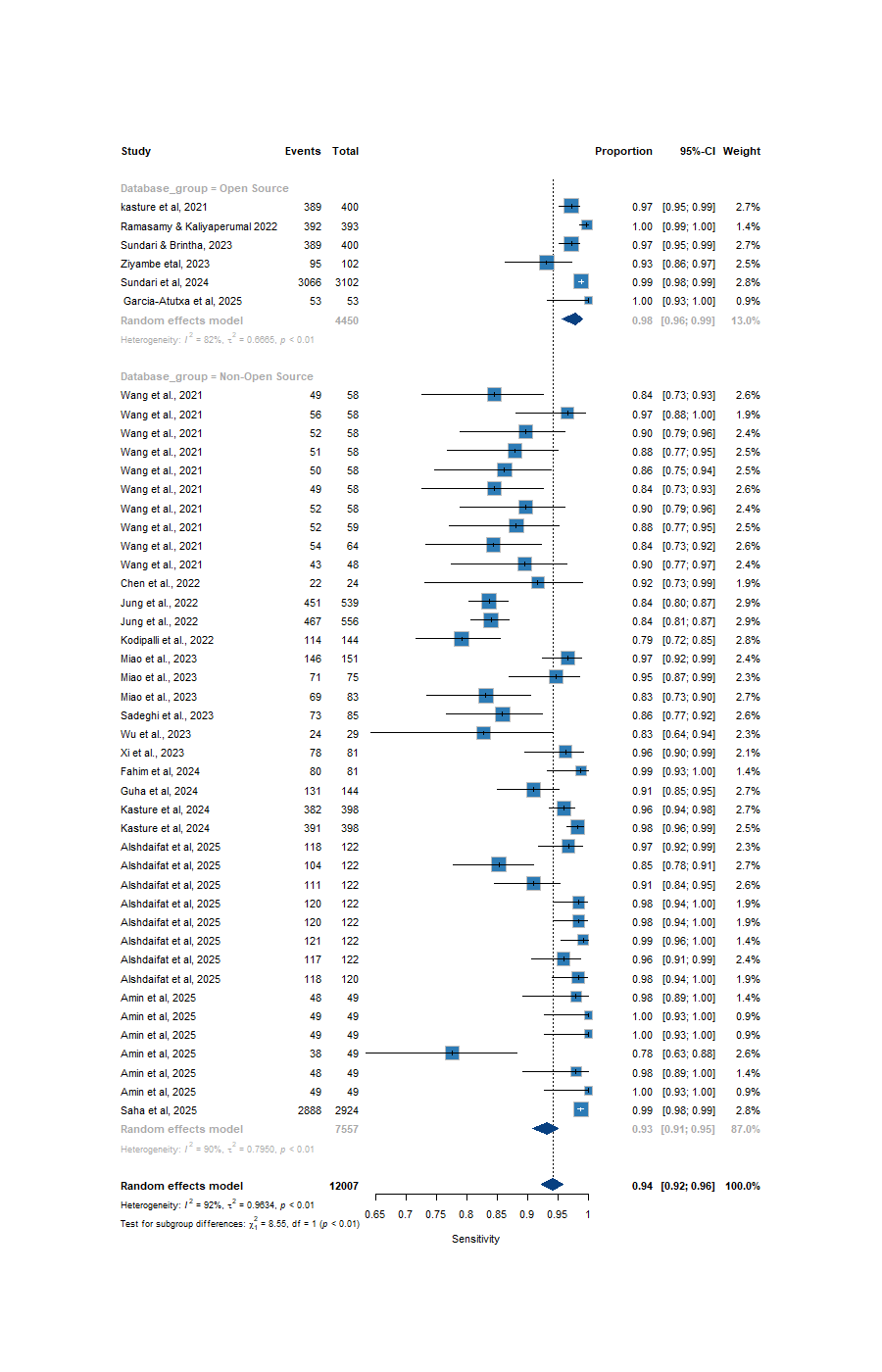 | 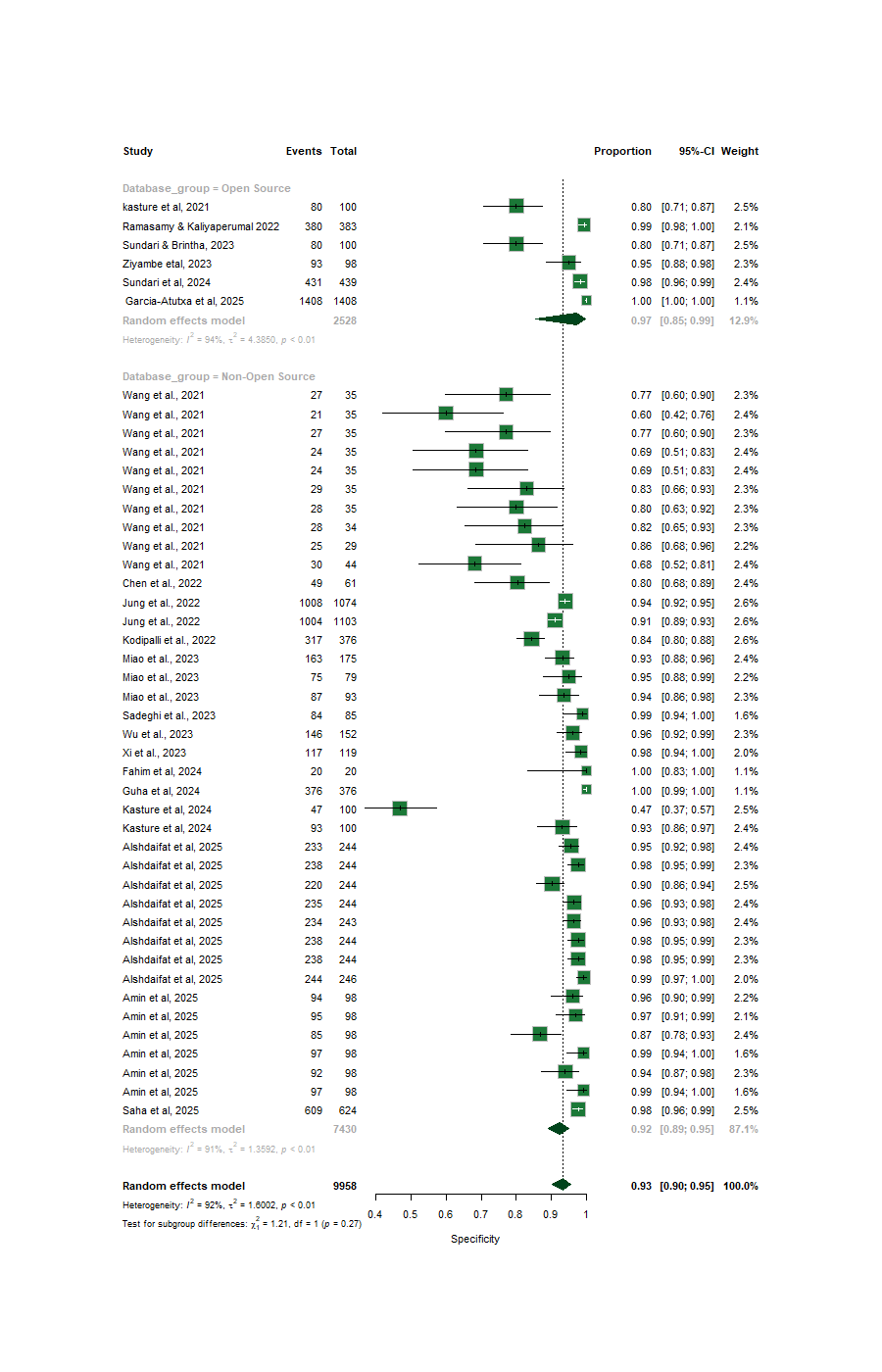 |
| **Figure S4a:** Sensitvity of studies used open source vs. non open source databases(20 studies, 45 tables). | **Figure S4b:** Specificity of studies used open source vs. non open source databases(20 studies, 45 tables). |

|

| Supplementary Table 1. Multivariate meta-regression result random-effects meta-regression Number of observations = 45  Method: Restricted maximum likelihood | | | | | | |
| --- | --- | --- | --- | --- | --- | --- |
| Covariate / Model Info | **Coefficient** | **SE** | **z-value** | **p-value** | **95% CI (Lower–Upper)** | **Significance** |
| Model Info: τ² = 4.9925, Wald χ²(7) = 14.9628, R² ≈ 0.1277, p = 0.0365 | – | – | – | – | – | – |
| Intrcpt | 4.2411 | 1.3174 | 3.2193 | 0.0013 | 1.6590 – 6.8231 | ** |
| ImagingModalities2 (CT) | 0.7178 | 1.7461 | 0.4111 | 0.6810 | -2.7045 – 4.1402 |  |
| ImagingModalities3 (MRI) | 1.2791 | 2.6194 | 0.4883 | 0.6253 | -3.8549 – 6.4131 |  |
| ImagingModalities4 (US) | 0.2311 | 1.4891 | 0.1552 | 0.8767 | -2.6874 – 3.1496 |  |
| DatasetTypeOpen (Open) | 2.1930 | 1.5137 | 1.4488 | 0.1474 | -0.7737 – 5.1598 |  |
| LearningType2 (Transfer) | 0.3282 | 0.3464 | 0.9472 | 0.3435 | -0.3509 – 1.0072 |  |
| Algorithm2 (DenseNet) | 0.4599 | 0.4431 | 1.0380 | 0.2993 | -0.4085 – 1.3283 |  |
| Algorithm3 (Other) | 0.9873 | 0.3035 | 3.2534 | 0.0011 | 0.3925 – 1.5821 | ** |
| Signif. codes: 0 ‘***’ 0.001 ‘**’ 0.01 ‘*’ 0.05 ‘.’ 0.1 ‘ ’ 1 | | | | | | |

Multivariate meta-regression was conducted using a random-effects model (REML) on 20 studies (k=45). Substantial residual heterogeneity was observed among studies (τ² = 4.993, I² ≈ 91%), supporting the use of a random-effects framework. Significant predictors included the “Other” algorithm category, which was associated with a positive effect on logDOR. In contrast, imaging modalities (CT, MRI, US), dataset type (Open vs Non-open), and Transfer learning were not significantly associated with logDOR. Overall, the included moderators explained 12.77% of the between-study variance, indicating that additional unmeasured factors may contribute to the observed heterogeneity (Supplementary Table 1).

| Supplementary Table 2: Multivariate meta-regression result random-effects meta-regression (Large Studies, k = 9) | | | | | | |
| --- | --- | --- | --- | --- | --- | --- |
| Covariate / Model Info | **Coefficient** | **SE** | **z-value** | **p-value** | **95% CI (Lower–Upper)** | **Significance** |
| Model Info | τ² = 5.8637 | – | – | – | QE(df=3)=74.18, p<0.0001 | QM(df=5)=4.89, p=0.429 |
| Intercept | 6.9632 | 7.9749 | 0.8731 | 0.3826 | -8.6673 – 22.5937 | – |
| ImagingModality4 (US) | -1.5491 | 4.5648 | -0.3394 | 0.7343 | -10.4958 – 7.3977 | – |
| DatasetTypeOpen | 0.9372 | 3.4574 | 0.2711 | 0.7863 | -5.8392 – 7.7135 | – |
| LearningType2 | 4.0898 | 3.4491 | 1.1857 | 0.2357 | -2.6704 – 10.8500 | – |
| AlgorithmFamily3 (Other) | 2.3554 | 4.5736 | 0.5150 | 0.6065 | -6.6086 – 11.3195 | – |
| TaskType2 | -5.3654 | 3.5827 | -1.4976 | 0.1342 | -12.3874 – 1.6565 | – |

Multivariate meta-regression was performed on 9 effect sizes derived from large studies (all with sample size ≥ 300). Substantial residual heterogeneity was observed (τ² = 5.86; QE p < 0.001), supporting the use of a random-effects model. However, the overall test of moderators was not statistically significant (QM(df = 5) = 4.89, p = 0.429), indicating that imaging modality, dataset type (open vs. non-open), learning strategy (fully trained vs. transfer learning), CNN architecture, and task type were not significantly associated with log diagnostic odds ratio (logDOR) in large studies (all p > 0.05). These findings suggest that, among large-sample studies, the examined methodological and algorithmic factors did not significantly explain between-study variability, and substantial unexplained heterogeneity remains.

| Supplementary Table 3. Leave-One-Out Sensitivity Analysis | | |
| --- | --- | --- |
| Study Removed | **Intercept Estimate** | **Intercept SE** |
| Kasture et al, 2021 | 4.3552 | 1.3035 |
| Wang et al., 2021 | 3.4737 | 1.6679 |
| Chen et al., 2022 | 4.2711 | 1.3539 |
| Jung et al., 2022 | 4.2545 | 1.3556 |
| Kodipalli et al., 2022 | 4.2102 | 1.2297 |
| Miao et al., 2023 | 4.2166 | 1.3660 |
| Ramasamy & Kaliyaperumal, 2022 | 4.1541 | 1.2777 |
| Sundari & Brintha, 2023 | 4.3210 | 1.3343 |
| Sadeghi et al., 2023 | 4.2142 | 1.3505 |
| Wu et al., 2023 | 4.2339 | 1.3660 |
| Xi et al., 2023 | 4.1748 | 1.3164 |
| Ziyambe et al., 2023 | 4.2997 | 1.3158 |
| Fahim et al, 2024 | 3.6710 | 1.4865 |
| Guha et al, 2024 | 4.2448 | 1.2649 |
| Kasture et al, 2024 | 4.8520 | 1.6135 |
| Sundari et al, 2024 | 4.1665 | 1.3302 |
| Alshdaifat et al, 2025 | 5.0293 | 1.3692 |
| Amin et al, 2025 | 4.2791 | 1.3204 |
| Garcia-Atutxa et al, 2025 | 5.2811 | 1.3378 |
| Saha et al, 2025 | 3.0968 | 1.5221 |


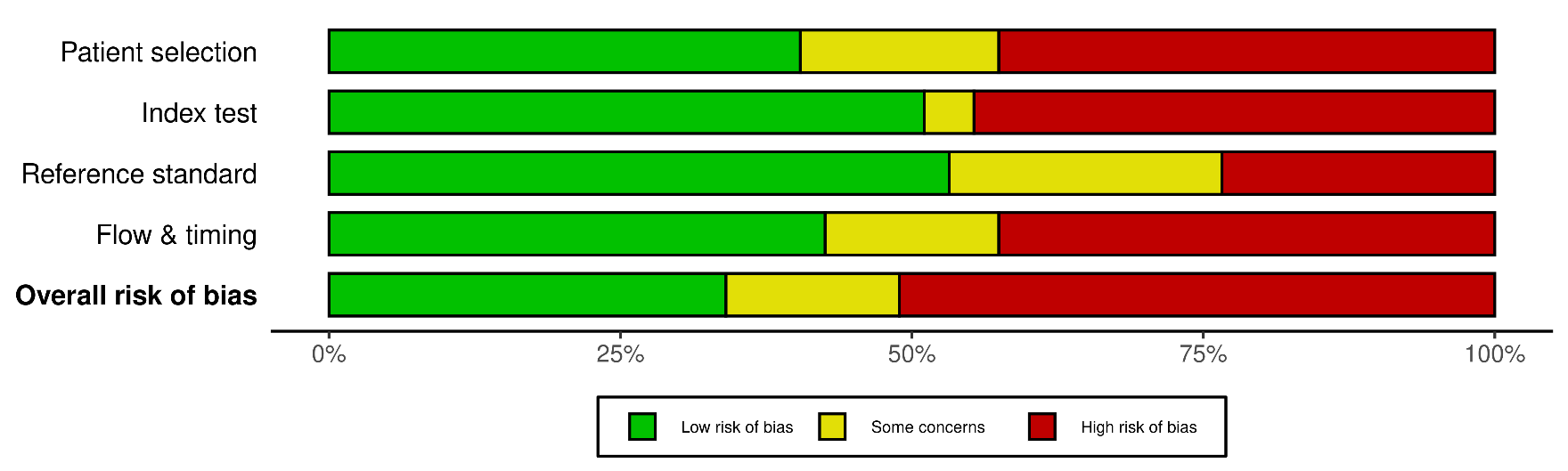


**Supplementary Figure 5a**: QUADAS-2 summary bar plot showing the percentages of studies with low, unclear, and high risk of bias across the 47 included studies in systematic review

**Supplementary Figure 5b**: QUADAS-2 summary showing risk of bias for each item in the included in systematic review studies presented as a traffic light plot.


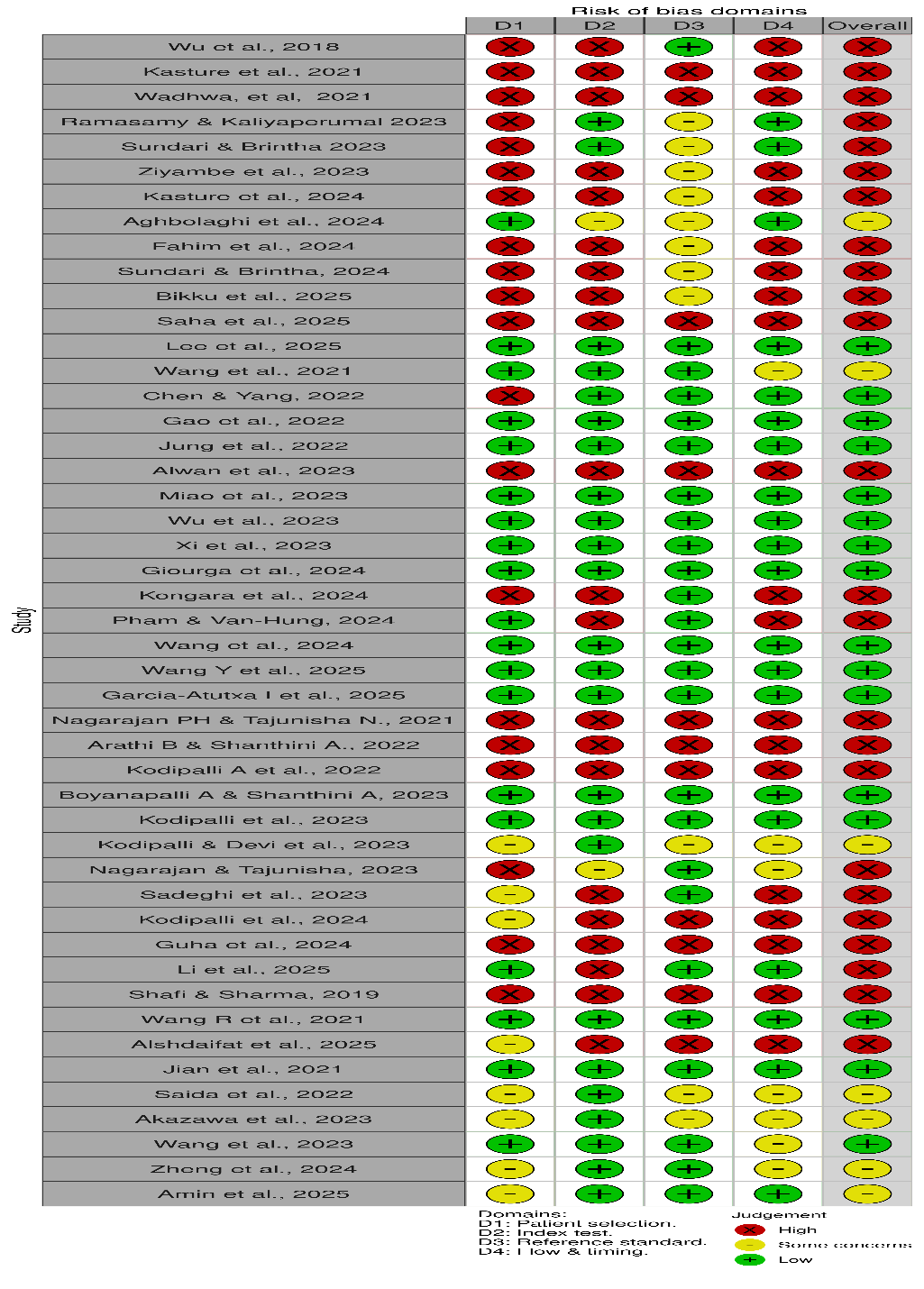


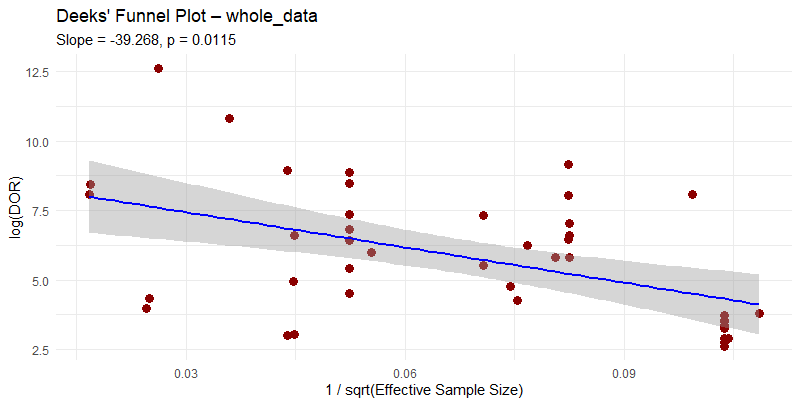


**Supplementary Figure S6**. Funnel plots suggested there was no publication bias

| **Table 4** Study-level characteristics and 2×2 diagnostic data of included studies. | | | | | | | | | | | |
| --- | --- | --- | --- | --- | --- | --- | --- | --- | --- | --- | --- |
| **StudyID** | **number studies** | **Imaging modalities** | **Algorithm (s)** | **Detection vs. Classification-text** | **Classification** | **Transfer/fully** | **TP** | **FN** | **FP** | **TN** | **Open vs. Non** |
| kasture et al, 2021 | 1 | Histopathological images | DCNN | Classification | Malignant vs. non-malignant | Fully learning | 389 | 11 | 20 | 80 | Open source |
| Wang et al., 2021 | 2 | Ultrasound image | VGG16 | Classification | Malignant vs. non-malignant | Fully learning | 49 | 9 | 8 | 27 | Non open source |
| Wang et al., 2021 | 2 | Ultrasound image | GoogLeNet | Classification | Malignant vs. non-malignant | Fully learning | 56 | 2 | 14 | 21 | Non open source |
| Wang et al., 2021 | 2 | Ultrasound image | ResNet34 | Classification | Malignant vs. non-malignant | Fully learning | 52 | 6 | 8 | 27 | Non open source |
| Wang et al., 2021 | 2 | Ultrasound image | MobileNet | Classification | Malignant vs. non-malignant | Fully learning | 51 | 7 | 11 | 24 | Non open source |
| Wang et al., 2021 | 2 | Ultrasound image | DenseNet | Classification | Malignant vs. non-malignant | Fully learning | 50 | 8 | 11 | 24 | Non open source |
| Wang et al., 2021 | 2 | Ultrasound image | VGG16 | Classification | Malignant vs. non-malignant | Transfer learning | 49 | 9 | 6 | 29 | Non open source |
| Wang et al., 2021 | 2 | Ultrasound image | GoogLeNet | Classification | Malignant vs. non-malignant | Transfer learning | 52 | 6 | 7 | 28 | Non open source |
| Wang et al., 2021 | 2 | Ultrasound image | ResNet34 | Classification | Malignant vs. non-malignant | Transfer learning | 52 | 7 | 6 | 28 | Non open source |
| Wang et al., 2021 | 2 | Ultrasound image | MobileNet | Classification | Malignant vs. non-malignant | Transfer learning | 54 | 10 | 4 | 25 | Non open source |
| Wang et al., 2021 | 2 | Ultrasound image | DenseNet | Classification | Malignant vs. non-malignant | Transfer learning | 43 | 5 | 14 | 30 | Non open source |
| Chen et al., 2022 | 3 | Ultrasound image | ResNet | Classification | Malignant vs. non-malignant | Transfer learning | 22 | 2 | 12 | 49 | Non open source |
| Jung et al., 2022 | 4 | Ultrasound image | DenseNet121 | Classification | Malignant vs. non-malignant | Transfer learning | 451 | 88 | 66 | 1008 | Non open source |
| Jung et al., 2022 | 4 | Ultrasound image | DenseNet161 | Classification | Malignant vs. non-malignant | Transfer learning | 467 | 89 | 99 | 1004 | Non open source |
| Kodipalli et al., 2022 | 5 | Computed tomography | Hybrid (Inception-ResNet v2) | Detection | Malignant vs. non-malignant | Transfer learning | 114 | 30 | 59 | 317 | Non open source |
| Miao et al., 2023 | 6 | Ultrasound image | ResNet | Detection | Malignant vs. non-malignant | Transfer learning | 146 | 5 | 12 | 163 | Non open source |
| Miao et al., 2023 | 6 | Ultrasound image | ResNet | Detection | Malignant vs. non-malignant | Transfer learning | 71 | 4 | 4 | 75 | Non open source |
| Miao et al., 2023 | 6 | Ultrasound image | ResNet | Detection | Malignant vs. non-malignant | Transfer learning | 69 | 14 | 6 | 87 | Non open source |
| Ramasamy & Kaliyaperumal 2022 | 7 | Histopathological images | DFCNN | Detection | Malignant vs. non-malignant | Fully learning | 392 | 1 | 3 | 380 | Open source |
| Sundari & Brintha, 2023 | 8 | Histopathological images | ResNet | Classification | Malignant vs. non-malignant | Transfer learning | 389 | 11 | 20 | 80 | Open source |
| Sadeghi et al., 2023 | 9 | Computed tomography | 3D CNN based on ResNet-50 | Detection | Malignant vs. non-malignant | Fully learning | 73 | 12 | 1 | 84 | Non open source |
| Wu et al., 2023 | 10 | Ultrasound image | ResNext50 | Classification | Malignant vs. non-malignant | Transfer learning | 24 | 5 | 6 | 146 | Non open source |
| Xi et al., 2023 | 11 | Ultrasound image | DenseNet | Detection | Malignant vs. non-malignant | Fully learning | 78 | 3 | 2 | 117 | Non open source |
| Ziyambe etal, 2023 | 12 | Histopathological images | Hybrid (CNN & GoogleNet (V3)) | Detection | Malignant vs. non-malignant | Transfer learning | 95 | 7 | 5 | 93 | Open source |
| Fahim et al, 2024 | 13 | Histopathological images | OVANet (Ensemble CNN: VGG19 + InceptionV3 + SE + spatial attention) | Classification | Malignant vs. non-malignant | Transfer learning | 80 | 1 | 0 | 20 | Non open source |
| Guha et al, 2024 | 14 | Computed tomography | Enhanced ResNet50 (ResNet60) | Detection | Malignant vs. non-malignant | Transfer learning | 131 | 13 | 0 | 376 | Non open source |
| Kasture et al, 2024 | 15 | Histopathological images | AlexNet | Classification | Malignant vs. non-malignant | Fully learning | 382 | 16 | 53 | 47 | Non open source |
| Kasture et al, 2024 | 15 | Histopathological images | VGG-19 | Classification | Malignant vs. non-malignant | Fully learning | 391 | 7 | 7 | 93 | Non open source |
| Sundari et al, 2024 | 16 | Histopathological images | ResNet-50 | Classification | Malignant vs. non-malignant | Transfer learning | 3066 | 36 | 8 | 431 | Open source |
| Alshdaifat et al, 2025 | 17 | Computed tomography | VGG16 | Detection | Malignant vs. non-malignant | Transfer learning | 118 | 4 | 11 | 233 | Non open source |
| Alshdaifat et al, 2025 | 17 | Computed tomography | VGG19 | Detection | Malignant vs. non-malignant | Transfer learning | 104 | 18 | 6 | 238 | Non open source |
| Alshdaifat et al, 2025 | 17 | Computed tomography | ResNet50 | Detection | Malignant vs. non-malignant | Transfer learning | 111 | 11 | 24 | 220 | Non open source |
| Alshdaifat et al, 2025 | 17 | Computed tomography | InceptionV3 | Detection | Malignant vs. non-malignant | Transfer learning | 120 | 2 | 9 | 235 | Non open source |
| Alshdaifat et al, 2025 | 17 | Computed tomography | Xception | Detection | Malignant vs. non-malignant | Transfer learning | 120 | 2 | 9 | 234 | Non open source |
| Alshdaifat et al, 2025 | 17 | Computed tomography | NASNetMobile | Detection | Malignant vs. non-malignant | Transfer learning | 121 | 1 | 6 | 238 | Non open source |
| Alshdaifat et al, 2025 | 17 | Computed tomography | NASNetLarge | Detection | Malignant vs. non-malignant | Transfer learning | 117 | 5 | 6 | 238 | Non open source |
| Alshdaifat et al, 2025 | 17 | Computed tomography | Xception_ViT | Detection | Malignant vs. non-malignant | Transfer learning | 118 | 2 | 2 | 244 | Non open source |
| Amin et al, 2025 | 18 | Magnetic resonance imaging | DenseNet-121 | Detection | Malignant vs. non-malignant | Transfer learning | 48 | 1 | 4 | 94 | Non open source |
| Amin et al, 2025 | 18 | Magnetic resonance imaging | VGG16 | Detection | Malignant vs. non-malignant | Transfer learning | 49 | 0 | 3 | 95 | Non open source |
| Amin et al, 2025 | 18 | Magnetic resonance imaging | VGG19 | Detection | Malignant vs. non-malignant | Transfer learning | 49 | 0 | 13 | 85 | Non open source |
| Amin et al, 2025 | 18 | Magnetic resonance imaging | ResNet50 | Detection | Malignant vs. non-malignant | Transfer learning | 38 | 11 | 1 | 97 | Non open source |
| Amin et al, 2025 | 18 | Magnetic resonance imaging | InceptionV3 | Detection | Malignant vs. non-malignant | Transfer learning | 48 | 1 | 6 | 92 | Non open source |
| Amin et al, 2025 | 18 | Magnetic resonance imaging | DenseNet121+GAM | Detection | Malignant vs. non-malignant | Transfer learning | 49 | 0 | 1 | 97 | Non open source |
| Garcia-Atutxa et al, 2025 | 19 | Ultrasound image | Hybrid CNN–Transformer model | Classification | Malignant vs. non-malignant | Transfer learning | 53 | 0 | 0 | 1408 | Open source |
| Saha et al, 2025 | 20 | Histopathological images | OvCan-FIND | Classification | Malignant vs. non-malignant | Transfer learning | 2888 | 36 | 15 | 609 | Non open source |
